# Integrin activation by the lipid molecule 25-hydroxycholesterol induces a proinflammatory response

**DOI:** 10.1038/s41467-019-09453-x

**Published:** 2019-04-01

**Authors:** Swechha M. Pokharel, Niraj K. Shil, Jeevan B. GC, Zachary T. Colburn, Su-Yu Tsai, Jesus A. Segovia, Te-Hung Chang, Smarajit Bandyopadhyay, Senthil Natesan, Jonathan C. R. Jones, Santanu Bose

**Affiliations:** 1 Department of Veterinary Microbiology and Pathology, Washington State University, Pullman, WA 99163 USA; 2 Department of Pharmaceutical Sciences, College of Pharmacy and Pharmaceuticals Sciences, Washington State University, Spokane, WA 99210 USA; 3 School of Molecular Biosciences, Washington State University, Pullman, WA 99163 USA; 4 Department of Microbiology and Immunology, The University of Texas Health Science Center at San Antonio, San Antonio, TX 78229 USA; 5 Molecular Biotechnology Core Laboratory, Lerner Research Institute, Cleveland Clinic, Cleveland, OH 44195 USA

## Abstract

Integrins are components of cell-matrix adhesions, and function as scaffolds for various signal transduction pathways. So far no lipid ligand for integrin has been reported. Here we show that a lipid, oxysterol 25-hydroxycholesterol (25HC), directly binds to α5β1 and αvβ3 integrins to activate integrin-focal adhesion kinase (FAK) signaling. Treatment of macrophages and epithelial cells with 25HC results in an increase in activated αvβ3 integrin in podosome and focal adhesion matrix adhesion sites. Moreover, activation of pattern recognition receptor on macrophages induces secretion of 25HC, triggering integrin signaling and the production of proinflammatory cytokines such as TNF and IL-6. Thus, the lipid molecule 25HC is a physiologically relevant activator of integrins and is involved in positively regulating proinflammatory responses. Our data suggest that extracellular 25HC links innate immune inflammatory response with integrin signaling.

## Abstract (web summary)

Integrins are key modulators of cell adhesion and signaling. Here the authors show, unexpectedly, that a complex of integrins and the lipid 25-hydroxycholesterol induces FAK activation and proinflammatory cytokine production, thereby serving as an integral part of innate immunity regulation.

## Introduction

Integrins, heterodimeric transmembrane receptors composed of one α- and one β-subunit, regulate numerous biological processes, including extracellular matrix assembly, cell adhesion, and cell migration^1–3^. In conjunction with a variety of associated proteins, integrin heterodimers function as signaling hubs, mediating both inside-out and outside-in signal transduction^3–5^. The ability of an integrin to signal depends on its conformational state^6–10^. Integrins cluster, forming a variety of matrix attachment sites, including focal adhesions (FAs) and/or podosomes^11^. FAs and podosomes contain many proteins, tether the cell to the extracellular matrix, function as membrane attachment sites for the actin cytoskeleton, are involved in cell motility and invasion, and act to scaffold integrin-mediated signaling events^12^. The latter are involved in numerous pathways, some of which lead to changes in gene expression via the actions of transcription factors such as MAPK and NFκB which, in turn, regulate various cellular functions, including the proinflammatory response and inflammation during innate immunity, the subject of this study^12^.

The innate immune system is an important host defense against pathogens (viruses, bacteria, fungi, and parasites), is also involved in the pathogenesis of various “non-infectious” inflammatory diseases, and depends, at least in part, on pattern recognition receptor (PRR) activation by pathogen associated molecular patterns (PAMPs)^13^. PRRs are expressed by cells of the innate immune system, including macrophages and certain epithelial cells. PRR activation by PAMPs represents the sentinel cellular mechanism triggering innate immunity and inflammatory response during infection. Nucleotide-binding oligomerization domain-containing protein 2 (Nod2) is a cytosolic PRR involved in innate immune inflammatory response during infection by viruses and bacteria and its hallmark function is to activate the NFκB signaling pathway, which promotes expression and production of a proinflammatory cytokine network^14–21^.

Numerous integrin ligands have been identified, including components of the extracellular matrix, counter-receptors on the surface of adjacent cells, certain growth factors, and members of the ADAM (a disintegrin and metalloproteinase) protein family^22,23^. However, a lipid ligand for integrins has not been reported.

In the current study, we identify 25-hydroxycholesterol (25HC), an oxygenated metabolite of cholesterol (oxysterol) catalyzed by the enzyme cholesterol 25-hydroxylase (C25H) as a lipid ligand of integrins. 25HC directly interacts with integrins to trigger focal adhesion kinase (FAK) activation. In addition, we identify the 25HC-related signaling network involved in optimizing the proinflammatory response following activation of the PRR Nod2. Our data, thus, show that extracellular 25HC, released from PRR-activated cells, is a molecular link bridging the PRR pathway and integrin-FAK signaling.

## Results

### 25HC activates FAK signaling

**Fig. 1:**
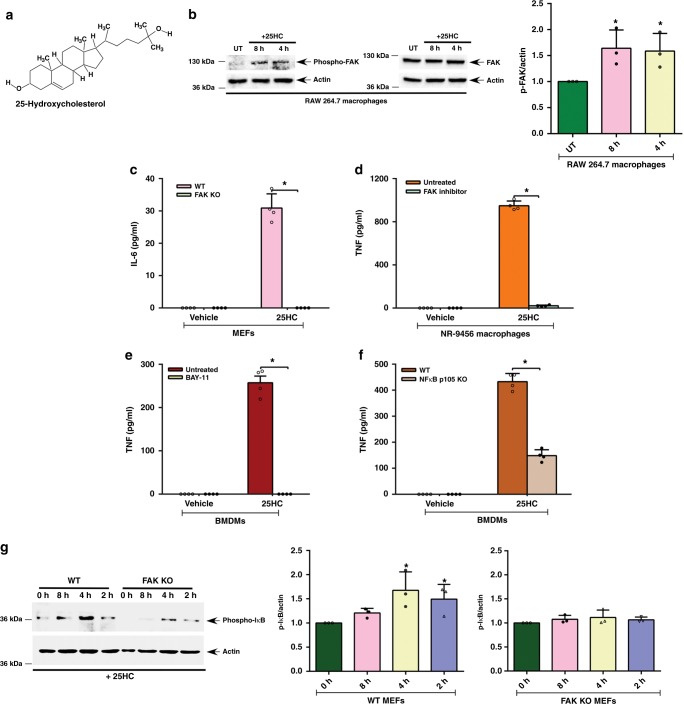
FAK-NFκB pathway is required for 25HC-dependent proinflammatory response. **a** Structure of 25-hydroxycholesterol (25HC). **b**–**g** Cells were treated with 50 µM 25HC for 8 h. FAK and NFκB activation and proinflammatory response in treated cells were evaluated. **b** Western blot and densitometric analyses of FAK activation (phospho-FAK, Tyr925) status in 25HC-treated RAW 264.7 macrophages. **c** IL-6 secretion from wild-type (WT) and FAK knockout (KO) MEFs (mouse embryo fibroblasts) treated with 25HC. **d** TNF secretion from NR-9456 macrophages treated with 25HC in the absence or presence of FAK inhibitor (5 µM). **e** TNF secretion from BMDMs treated with 25HC in the absence or presence of NFκB inhibitor Bay 11-7082 (BAY-11) (10 µM). **f** TNF secretion from WT and NFκB p105 KO BMDMs treated with 25HC. **g** Western blot and densitometric analyses of phospho-IκB status in 25HC-treated WT and FAK KO MEFs. The ELISA values (mean ± standard deviation) are representative from two or three independent experiments (*n* = 4). *p ≤ 0.05 using a Student’s *t-*test. The densitometric quantification values for phospho-FAK (p-FAK) and phospho-IκB (p-IκB) immunoblots represent the ratio of phospho-FAK:actin and phospho-IκB:actin and the fold-induction was calculated after normalizing with the control untreated (UT) or control 0 h group. The densitometric values represent the mean ± standard deviation from three independent studies. *p ≤ 0.05 using a Student’s *t-*test

25HC (Fig. 1a) is an oxygenated metabolite (oxysterol) of cholesterol catalyzed by the enzyme cholesterol 25-hydroxylase (C25H)^24,25^. A recent study provided evidence that soluble (extracellular) 25HC activates a proinflammatory response in macrophages however the mechanism by which it does so was not elucidated^26^. Nonetheless, intracellular signaling induced by extracellular 25HC is likely a consequence of its binding to a membrane signaling receptor. While there are a number of candidate receptors, previous reports demonstrating a role of integrin-FAK signaling in inducing proinflammatory response led us to hypothesize that activation of integrin-FAK signaling by 25HC may represent the molecular mechanism driving the proinflammatory activity of 25HC^27,28^. FAK is a key adaptor protein in integrin-mediated signal transduction pathways^1,2^. Therefore, we first investigated whether 25HC activates FAK and the role of activated FAK in mediating the 25HC-dependent proinflammatory response.

Phosphorylation of FAK (at tyrosine residues) is an indicator of its activation and is a consequence of integrin signaling^1,2^. Treatment of macrophages with 25HC triggered FAK phosphorylation as detected by western blot analyses (Fig. 1b). Densitometric analyses of the phospho-FAK western blot data revealed significant induction of FAK following 25HC treatment (Fig. 1b). We next assessed whether FAK activation played a role in 25HC-mediated induction of the proinflammatory response by assaying secretion of the proinflammatory cytokines TNF-α (TNF) and IL-6 in cells expressing FAK, as well as in cells in which FAK was absent or inhibited. We focused our studies on these two cytokines as they are critical proinflammatory mediators that shape innate inflammatory responses during various infectious (e.g., viral, bacterial) and non-infectious diseases/conditions such as arthritis. Surprisingly, there was a complete abrogation of IL-6 secretion in 25HC-treated FAK knockout (KO) mouse embryo fibroblasts (MEFs) (Fig. 1c). Moreover, the inhibition of FAK activity in macrophages drastically reduced the 25HC-induced production of both TNF and IL-6 (Fig. 1d and Supplementary Fig. 1a). Lactate dehydrogenase (LDH) cytotoxicity assays revealed negligible loss of cell viability in 25HC-treated FAK KO macrophages or macrophages treated with a FAK inhibitor (Supplementary Table 1).

As FAK signaling activated a number of downstream molecules, including NFκB, we evaluated whether NFκB mediates the cellular response to 25HC treatment. BAY-11, an NFκB inhibitor, reduced TNF and IL-6 secretion from macrophages treated with 25HC (Fig. 1e and Supplementary Fig. 1b). This result was further validated as TNF production was reduced by 66% in 25HC-treated NFκB KO (NFκB p105 KO) primary bone marrow-derived macrophages (BMDMs) (Fig. 1f). Accordingly, NFκB activation was diminished in 25HC-treated FAK KO cells, evaluated by western blot to assess the levels of phosphorylated IκB (phospho-IκB) in FAK KO cells (Fig. 1g). Densitometric analyses of the phospho-IκB western blot data revealed significant induction of NFκB (i.e., increased phospho-IκB levels) in 25HC-treated wild-type (WT) cells, but not in FAK KO cells (Fig. 1g). Thus, our studies have identified a previously unknown signal transduction network in which 25HC activates FAK-NFκB signaling.

### 25HC binds integrins α5β1 and αvβ3

**Fig. 2:**
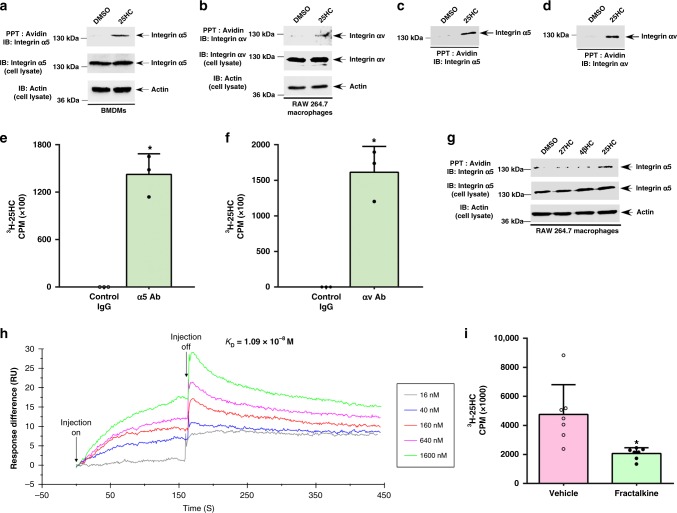
25HC interacts with α5β1 and αvβ3 integrins. **a**, **b** Biotinylation reactions were performed in the presence of either 25HC or DMSO (control). Cell lysates collected from BMDMs (**a**) or RAW 264.7 macrophages (**b**) incubated with biotinylated 25HC (biotin-25HC) or DMSO at 4 °C were precipitated (PPT) with avidin–agarose and subsequently immuno-blotted (IB) with either α5 integrin (**a**) or αv integrin (**b**) antibody. Total lysates were also blotted with α5 integrin (**a**), αv integrin (**b**), and actin (**a**, **b**) antibodies. **c**, **d** Biotin-25HC and DMSO (control) were incubated (PPT; precipitated) with avidin–agarose beads. 25HC conjugated beads were then incubated either with purified α5β1 integrin (**c**) or αvβ3 integrin (**d**). The avidin–agarose bound complex was subsequently immuno-blotted with either α5 integrin (**c**) or αv integrin (**d**) antibody. **e**, **f** Protein G-agarose beads were conjugated with either control IgG or antibody (Ab) specific for α5 integrin (**e**) and αv integrin (**f**). The beads were then incubated with either purified α5β1 integrin (**e**) or αvβ3 integrin (**f**) protein. Control beads (control IgG + purified integrin protein) and integrin protein bound beads (integrin-specific antibody + purified integrin protein) was incubated with tritiated ^3^H-25HC. Bead-bound radioactivity (count per minute; CPM) was measured by scintillation counter. **g** Cell lysate collected from RAW 264.7 macrophages incubated with biotin-25HC, biotinylated 27-hydroxycholesterol (biotin-27HC), biotinylated 4β-hydroxycholesterol (biotin-4βHC) or DMSO (control) at 4 °C was precipitated (PPT) with avidin–agarose and subsequently immuno-blotted with α5 integrin antibody. Total cell lysate was also blotted with α5 integrin and β-actin antibodies. **h** Surface plasmon resonance (SPR) analyses were performed by injecting increasing concentrations of 25HC (16 nM, 40 nM, 160 nM, 640 nM, 1.6 µM) into both the αvβ3 integrin-immobilized and control surface of the CM5 chips. Each background-corrected sensorgram is a representative of replicate runs. **i** Binding of radioactive ^3^H-25HC to immobilized αvβ3 integrin protein in the presence or absence of purified chemokine domain of fractalkine protein was assessed as described in the “methods” section. Immunoblot images are representative from three independent experiments. The radioactive values (mean ± standard deviation) are representative from two independent experiments. *p ≤ 0.05 using a Student’s *t*-test

As integrin activation triggers FAK activation^1,2^, we next assayed whether 25HC associates with integrins. To that end, we generated biotin labeled 25HC (biotinylated 25HC or biotin-25HC) and confirmed the latter was indeed biotinylated and active in triggering proinflammatory response (Supplementary Fig. 2a, b). We incubated chilled BMDMs with biotin-25HC (at 4 °C) to promote 25HC interaction with cell surface moieties in the absence of their internalization. Following incubation, protein complexes were precipitated with avidin–agarose and subjected to western blot analyses with α5 integrin antibody. 25HC, but not DMSO treatment, precipitated α5 integrin, indicating an association between 25HC and α5 integrin subunit-containing complexes on the cell surface (Fig. 2a). A similar experiment with chilled RAW 264.7 macrophages also revealed interaction of biotin-25HC with cell surface αv integrin subunits (Fig. 2b).

Next, in vitro binding assays were performed to examine whether 25HC directly interacts with purified integrin heterodimers. Biotin-25HC conjugated to avidin–agarose beads was incubated with either purified α5β1 or αvβ3 integrin (Supplementary Fig. 2c) and the complex was subjected to western blotting with either α5 or αv integrin antibodies. Our studies revealed direct association of 25HC with both α5β1 and αvβ3 integrins (Fig. 2c, d). Finally, we corroborated these findings using tritiated-25HC (^3^H-25HC). ^3^H-25HC was active as it triggered a proinflammatory response in macrophages (Supplementary Fig. 2d). Protein G-agarose beads were conjugated with either control IgG or antibody specific for α5 and αv integrin heterodimers, which were subsequently incubated with purified α5β1 or αvβ3 integrin, respectively. Control beads and integrin protein-bound beads were then incubated with ^3^H-25HC. We detected binding of radioactive 25HC to both purified α5β1 and αvβ3 integrins (Fig. 2e, f). The interaction of 25HC with integrin was specific as our in vivo studies revealed that two closely related oxysterols, 27-hydroxycholesterol (27HC) and 4β-hydroxycholesterol (4βHC), failed to interact with cell surface α5 integrin subunits, in contrast to 25HC (Fig. 2g).

**Fig. 3:**
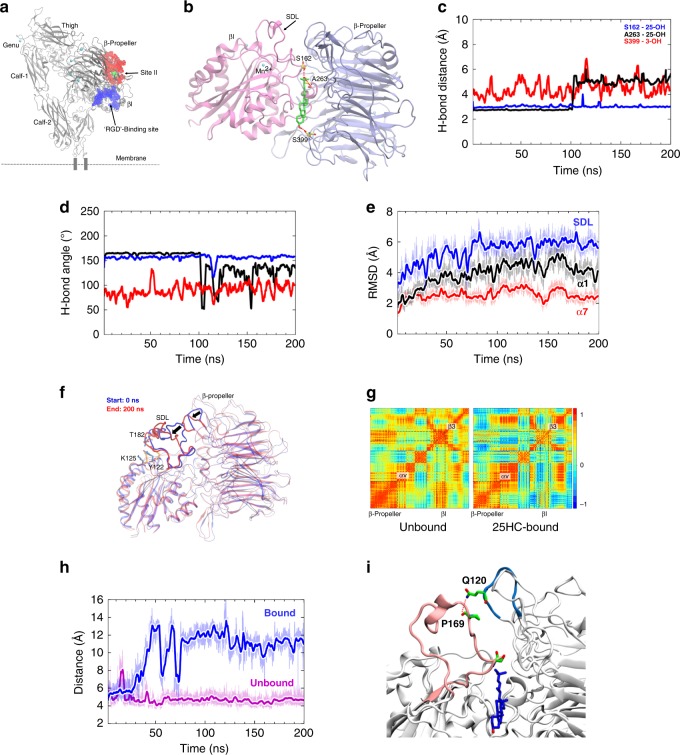
Modeling identifies specific interactions between αvβ3 integrin and 25HC. **a** Ectodomain structure of αvβ3 integrin containing ‘RGD’’-binding site (site I) (blue) and site-II (red) residues highlighted by a layer of solvent molecule of radius 1.4 Å. The site-II-bound 25HC molecule is presented as a surface model in green and Mn^2+^ ions from metal-ion-dependent ligand-binding site (MIDAS) and adjacent to MIDAS (ADMIDAS) sites of βI domain, β-propeller domain and genu knee region are presented in cyan. **b** Potential interacting residues for molecular recognition of αvβ3 integrin by 25HC were identified in a docking simulation. H-bonds are highlighted in dashed lines in red. **c**, **d** Ser399 of β-propeller (red), Ser162 (blue) and Ala263 (black) of βI domain make stable H-bond interactions with 3- and 25-hydroxyl groups of 25HC, respectively. The H-bond distances (**c**) and H-bond angles (**d**) are plotted against MD simulation time (200 ns). **e** The root-mean-square deviations (RMSD) measured for specificity-determining loop (SDL), α1- and α7 helices during the entire 200 ns long simulation. The SDL region undergoes significant conformational change (RMSD of ~6 Å) upon 25HC binding. **f** The conformational change in the SDL is accompanied by disruption of H-bond interaction between Tyr122-Thr182 residues, as well as change in the β-propeller blade that interacts with SDL. Movement of the regions from the beginning 0 ns (blue) to the end 200 ns (red) is indicated by arrows. **g** Correlation matrices (as heat maps) indicating correlated (red) or anti-correlated motions (blue) of α- and β-subunits of αvβ3 integrin in unbound and 25HC-bound states. **h**, **i** The distance between the amide NH of Q120 of the β-propeller domain and backbone carbonyl oxygen of P169 of the SDL. Breakage of this electrostatic interaction in the 25HC-bound protein (blue) during the simulation, around ~30 ns simulation causes significant increase in the distance, which remain intact in the unbound protein (magenta) during the entire 200 ns simulation time

We further confirmed the specificity of 25HC–integrin interaction by two distinct methods, (a) surface plasmon resonance (SPR), and (b) competitive binding assay. In these two studies we analyzed the interaction of 25HC with αvβ3 integrin, as αvβ3 integrin interacts with 25HC (Fig. 2b, d, f) and, more importantly, our molecular dynamics (MD) study (Fig. 3, below) suggested specific binding of 25HC to site-II of αvβ3 integrin. First we performed SPR analyses by using a sensor chip in which αvβ3 integrin protein was immobilized. The specificity of 25HC–integrin interaction was borne out by the observation that 25HC bound to αvβ3 integrin at a high-affinity (equilibrium dissociation constant or *K*_D_ of 1.09 × 10^−8^ M or 10.9 nM) (Fig. 2h). Interestingly, the *K*_D_ value of 25HC-intigrin was comparable to the SPR-derived *K*_D_ values reported for binding of integrin molecules with its matrix and growth factor ligands, including fibronectin [*K*_D_ = 4.4 nM for α5β1-fibronectin binding^29^], collagen-I [*K*_D_ = 24 nM for α1β1-collagen binding^30^], vitronectin [*K*_D_ = 64 nM for αvβ3-vitronectin binding^31^], and insulin like growth factor-1 or IGF1 [*K*_D_ = 31 nM for αvβ3-IGF1 binding^32^]. Interestingly, 25HC-αvβ3 integrin affinity (Fig. 2h) was comparable to that of other molecules that interact with site-II of αvβ3 integrin, including phospholipase A2 (*K*_D_ = 211 nM for αvβ3-phospholipase A2 binding to site-II)^33^ and fractalkine (*K*_D_ = 0.3 nM–6.9 nM for αvβ3-fractalkine binding to site-II)^34^. Thus, our SPR results showing high-affinity binding of 25HC with integrin demonstrated that 25HC–intgerin interaction is highly specific.

We next performed a modified “ELISA-type” 25HC-integrin-binding assay^34–36^. As our MD studies (Fig. 3, below) revealed binding of 25HC to the site-II of αvβ3 integrin, we choose fractalkine protein as a competitor since previous studies have shown that the chemokine domain (CD) of fractalkine protein (fractalkine-CD) binds to the site-II of αvβ3 integrin^34,35^. Plates were coated with purified αvβ3 integrin protein followed by addition of tritiated-25HC (^3^H-25HC). In some experiments, immobilized αvβ3 integrin was first incubated with purified fractalkine-CD protein (~9 kDa) (Supplementary Fig. 2e) prior to addition of ^3^H-25HC. ^3^H-25HC bound to the αvβ3 integrin protein was then evaluated. Our result indicated a specific interaction of 25HC with site-II of the integrin molecule as there was a significant loss (>60%) of 25HC binding to αvβ3 integrin in the presence of fractalkine-CD protein (Fig. 2i). This result demonstrated specific interaction of 25HC with integrin molecules.

### Molecular modeling of 25HC–αvβ3 integrin interaction

Next, we performed molecular docking and MD simulations to interrogate the potential interactions and outside-in activation mechanism of 25HC with integrins using αvβ3 integrin–25HC complex as a model. MD simulation is a useful tool for understanding biological function-based conformational dynamics as it provides high-resolution details of spatial arrangement of atoms in a system over time. We investigated two possible binding sites for 25HC. The first was at site I, the RGD-binding site (Fig. 3a) and the second site (site-II) was the binding site for fractalkine (FKN) and phospholipase A2 (sPLA-IIA) (Fig. 3a and Supplementary Table 2)^35,36^. We performed an induced fit docking protocol in which the relative positions of side chains of binding site residues were optimized at the final refinement stage. The subsequent MD simulation revealed that 25HC did not bind to the RGD-binding site (Supplementary Movie 1). Rather, binding was favored at site-II (Supplementary Movie 2). The best pose from the docking simulation (Fig. 3b) was selected based on the number and strength of H-bond interactions (H-bond distance and angle), number of hydrophobic interactions, and buried surface area.

The ligand bound protein–25HC complex and the unbound protein were subjected to 200 ns MD simulations each and the resulting trajectories were analyzed for conformational changes in αvβ3 integrin upon 25HC binding. We focused on three H-bonds (Fig. 3b) observed between 3- and 25-OH groups of 25HC and binding site residues at the interface between βI and β-propeller domains. The residue A263 from the βI domain, situated at the buried interface and close to the specificity-determining loop (SDL) of the molecule, makes a strong H-bond between its backbone carbonyl oxygen and the 25-OH group of 25HC. This H-bond had just over 50% occupancy and was observed to have geometries (average distance ~2.8 Å and angle ~165°) that are indicative of strong H-bond interactions (Fig. 3c, d). At ~110 ns, the ligand readjusts its orientation (Fig. 3c, d) and move a little upward towards S162 of the SDL. Owing to this reorientation, the H-bond with A263 weakens but seems to continue as electrostatic interactions. The 25-OH group was also pinned by S162 from the SDL. The backbone amide –NH and sidechain –OH groups of this residue intermittently flipped sides and consistently engaged in H-bond interactions with optimal geometries (distance ~2.9–3.0 Å and angle ~158–160°) during the entire simulation with occupancy of >95%. Thus, conformational flipping of Ser162 generated sufficient stimulus to trigger SDL movement, leading to permanent conformational change (discussed below). This event occurred approximately at 30 ns of the 200 ns simulation. During the entire 200 ns simulation, the bonding partners engaged in the H-bond interaction showed random oscillation from the ideal geometries, indicating the bond dynamics (Fig. 3c, d and Supplementary Movie 2). The 3-OH group of the ligand engages in moderately strong electrostatic interaction with –OH sidechain of S399 throughout the entire 200 ns simulation often satisfying the criteria of H-bonds (Fig. 3c, d and Supplementary Movie 2). In addition, 25HC made several nonpolar interactions with residues at the interface of the β-propeller and βI domains (Y18, K42, W93, L111, and M400 of the β-propeller domain and V161, M165, A263, I265, Q267, V266, T285, and T286 of the βI domain).

The stable and lasting interactions of 25HC at the integrin-binding site [root-mean-square deviation (RMSD) <1 Å, Supplementary Fig. 3e] were partly due to the amount of hydrophobic surface buried upon ligand binding. It is well documented that large gains in binding free energy of several kcal/mol per heavy atom (non-hydrogen) can be obtained when a lipophilic protein pocket is optimally occupied by nonpolar ligand atoms^37^. It is noteworthy to mention that the aryl rings of W93 and Y18 from the β-propeller domain also engaged in Alkyl–Aryl interactions with the –CH3 groups of 25HC. The stable van der Waal (steric) interactions between these two interacting molecules can be qualitatively observed by the distance (Supplementary Fig. 3f) measured between the center of mass (COM) of binding site residues and that of 25HC molecule.

The SDL in the βI domain of the β3 subunit is formed by the residue sequence K156-G189 and has been shown to be critical for ligand binding at the ‘RGD’’-binding site^32,38,39^. In addition, the SDL determines α-subunit association specificity and the conformation of the SDL is dependent on the associating α-subunit^40^. In our 200 ns long MD simulation run, we observed that the H-bond network between K125, Y122, and T182 of SDL was disrupted by 25HC binding to αvβ3 integrin. This was not observed in the unbound state simulation. The disruption mainly occurred between T182-Y122 H-bond that connects the loop to the α1-helix (Fig. 3f). It is important to note that this disruption arose at exactly the same time (~30–40 ns) when the SDL underwent a significant conformational change (RMSD of >6 Å) (Fig. 3e and Supplementary Fig. 3a, c). The conformational change observed in the SDL is also caused by the disruption of notable electrostatic interaction between Q120 of the β-propeller and P169 of the SDL (Fig. 3h, i), which remains stable in the unbound protein. Thus, the 25HC-mediated change in this region could potentially alter the ligand-binding ability at the RGD-binding site (i.e., site I), as well as at binding sites on both the SDL and β-propeller (Fig. 3b, f). The movement of the SDL (Fig. 3f and Supplementary Fig. 3a, c) resulted in notable changes in the 2- and 3-coils of the propeller blades that interact with the βI domain. The overall change in the surface property resulted in an increase of >200 Å^2^ in the solvent accessible surface area (SASA) of these loop regions combined (Supplementary Fig. 3c). Additional essential dynamics analyses quantifying global conformational changes, as well as correlated motions of various subunits are presented as RMSD (Fig. 3e), RMSF (Supplementary Fig. 3a), correlation matrix (Fig. 3g), SASA (Supplementary Fig. 3b) and porcupine plot (Supplementary Fig. 3d). The detailed description of these analyses are provided as Supplementary Note 1.

Correlated motions in biological molecules are essential for their function, for example, during ligand-mediated allosteric signal transduction^41^. The correlated and anti-correlated motions among various domains of the integrin αvβ3 were visualized as porcupine plot and heat maps generated using Visual Molecular Dynamics (VMD 1.9.2) and Lattice package in R (version 3.3.1) software, respectively (Fig. 3g, Supplementary Fig. 3d, and Supplementary Movie 3). The porcupine plot revealed an interesting pattern of dynamics between the head and leg regions of the αvβ3 integrin, in which the ligand-binding site containing the β-propeller and βI domains made inward motion toward the calf-2 domain of the leg region, possibly transmitting the activation signal directly to the leg region attached to the transmembrane tail. The heat maps of correlation matrices depict the conformational and dynamic difference between unbound and 25HC-bound states (Fig. 3g). The increased anti-correlated motions correspond to the observed conformational change at the interface between β-propeller and SDL region of βI (dotted regions in the map in Fig. 3g), as well as notable conformational changes of the α1 and α7 helices (Fig. 3e and Supplementary Fig. 3a) in the molecule.

Binding of RGD motif-containing ligand mimetic compounds to site I is mechanically coupled to tertiary changes in the βA domain, involving inward movement of the N-terminal α1 helix toward the MIDAS, forcing reorganization of the loop between the C-terminal F strand and α7 helix, a one-turn displacement of helix α7 and a hybrid domain swing out^42^. We carefully monitored any conformational changes in these regions of site I, upon 25HC binding to site-II. The root-mean-square fluctuations (RMSF), RMSD, and SASA calculations revealed notable movement of the α1-helix (RMSD change of ~4.2 Å and SASA change of 300 to 500 Å for the entire βI domain), which stabilized after an ~40 ns simulation (Fig. 3e and Supplementary Fig. 3a, b). The angles between hybrid and βI domains, as well as between β-propeller and calf-2 domains were calculated between their respective centers of mass. The angle between the hybrid and βI domain showed a very moderate ~2° increase for the first 30 ns of simulation time, which eventually dropped to its original angle of ~82°. In contrast, unbound integrin exhibited a relatively larger increase (~6°) in the angle, which eventually dropped to the original angle as well. The angle between β-propeller and calf-2 domains exhibited notable movement in opposite directions, resulting in a decrease of angle, from ~46° to ~42° between the domains (Supplementary Fig. 3d and Supplementary Movie 3).

Our MD study indicated that 25HC binds to site-II of αvβ3 integrin and is consistent with both our SPR analyses (Fig. 2h) and competitive binding assay (Fig. 2i). Indeed, based on our data, we postulate that binding of 25HC to site-II of αvβ3 integrin may directly trigger integrin signaling as observed previously for ligands that bind to site-II. Furthermore, binding of 25HC to site-II of αvβ3 integrin generated significant conformational changes at the βI domain, mainly altering the shape of the SDL, resulting in corresponding changes at the interacting surface of β-propeller domain. The observed changes at both the SDL and coils/turns of the β-propeller could, therefore, result in functional alteration in ligand-binding affinity at the ‘RGD’’-binding site (i.e., site I), leading to binding of αvβ3 integrin-specific extracellular matrix (ECM) ligands to site I. Further MD stimulation and analyses also revealed binding of 25HC to the site-II of α5β1 integrin (Supplementary Notes).

### 25HC activates αvβ3 integrin at matrix adhesion sites

**Fig. 4:**
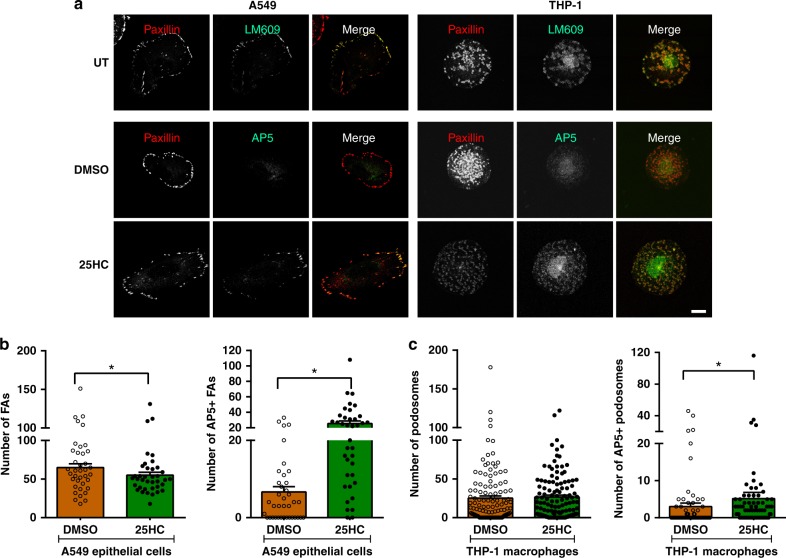
25HC activates αvβ3 integrin in epithelial cell focal adhesions and macrophage podosomes. **a** Human lung epithelial cells (A549 cells) or differentiated THP-1 macrophages were subjected to various treatments and immunostained as indicated. In untreated (UT) A549 and THP-1 cells (first row), αvβ3 integrin, immunostained using the antibody LM609, was present in paxillin-positive focal adhesions and podosomes, respectively. In contrast, active αvβ3 integrin staining, indicated using the antibody AP5 (second and third row), was found almost exclusively in cells treated for 2 h with 0.5 µM 25HC compared with DMSO-treated controls. Merged images show the co-localization of αvβ3 integrin (total or activated) with focal adhesions/podosomes. Bar, 10 µm. **b** Quantification of the number of focal adhesions (FAs) and AP5^+^ FAs in A549 cells (three experiments, >12 cells per experiment; *n* ≥ 30). **c** Quantification of the number of podosomes and AP5^+^ podosomes in THP-1 cells (three experiments, 25 cells per experiment; *n* ≥ 70). Two- and One-tailed Wilcoxon rank-sum tests were performed to evaluate differences between total and AP5^+^ FA/podosome counts, respectively. Graphs depict mean ± SEM. *p ≤ 0.05

Our MD studies indicated that 25HC binding to αvβ3 integrin resulted in changes in integrin conformation. As the ability of integrins to signal depends on their conformation, we tested the hypothesis that the binding of 25HC to αvβ3 integrin affects its activity. Initially, we used the human epithelial cell line A549 as A549 cells express αvβ3 integrin in their focal adhesions (FAs) (Fig. 4a, b). In addition, we chose these cells due to the availability of human-reactive antibody probes, including those that recognize integrin subunits in an active conformation. 25HC treatment of A549 cells induced only a small, 15%, decrease in the number of paxillin-positive FAs (Fig. 4b). However, when we immunostained control and 25HC-treated A549 cells with antibodies targeting paxillin and active αvβ3 integrin using the antibody AP5, we observed negligible levels of active αvβ3 integrin in the FAs of untreated cells but a dramatic and significant increase in active αvβ3 integrin was observed following 25HC treatment (Fig. 4a, b). It should be noted that treatment of cells with 25HC did not alter the internalization rate of cell surface αvβ3 integrin. The internalization rate of αvβ3 integrin was similar in DMSO versus 25HC-treated cells (Supplementary Fig. 4a).

25HC has been demonstrated to be an effector of innate immunity in macrophages^26^. Thus, we next assayed the impact of 25HC on αvβ3 integrin localization in human THP-1 monocytic cells that were induced to differentiate into a macrophage-like phenotype by phorbol 12-myristate 13-acetate (PMA). αvβ3 integrin co-localized with paxillin in the podosomes of PMA-differentiated THP-1 cells (Fig. 4a). Although 25HC treatment did not affect the number of podosomes in THP-1 cells, substantially more activated αvβ3 integrin was incorporated into the podosomes of 25HC-treated cells compared with their vehicle-treated counterparts (Fig. 4a, c).

As FAs and podosomes are involved in motility, we suspected that the activation of αvβ3 integrin in these structures would affect cell migration. However, 25HC treatment did not have a substantial impact on speed or directed migration (processivity) (Supplementary Fig. 4b, c). Rather, the consequences of 25HC-mediated αvβ3 integrin and FAK activation in FAs and podosomes may be restricted to the signaling properties of these structures, a possibility we evaluated next.

### 25HC triggers integrin-dependent proinflammatory response

**Fig. 5:**
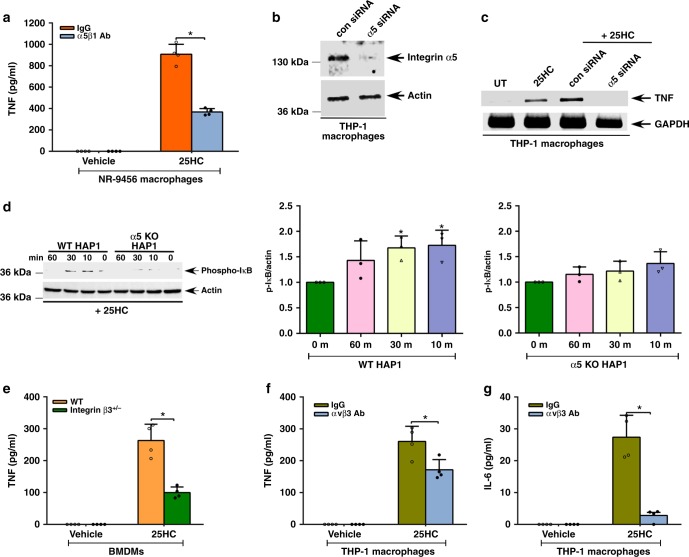
α5β1 and αvβ3 integrins regulate 25HC-mediated proinflammatory response. **a**, **c**–**g** α5β1 and αvβ3 integrin-deficient cells were treated with 50 µM 25HC for 8 h to evaluate NFκB activation and proinflammatory response. **a** TNF secretion from 25HC-treated NR-9456 macrophages in the presence of either control IgG or α5β1 integrin blocking antibody (Ab). **b** Western blot analyses of α5 integrin protein expression in THP-1 cells transfected with either control siRNA (con siRNA) or α5 integrin-specific siRNA (α5 siRNA). **c** RT-PCR analyses of TNF expression in 25HC-treated THP-1 cells transfected with either control siRNA (con siRNA) or α5 integrin-specific siRNA (α5 siRNA). **d** Western blot and densitometric analyses of phospho-IκB status in 25HC-treated wild-type (WT) and α5 integrin knockout (KO) HAP1 cells. **e** TNF secretion from 25HC-treated WT and β3 integrin-deficient (β3^+/−^ cells) BMDMs. **f**, **g** TNF (**f**) and IL-6 (**g**) secretion from 25HC-treated THP-1 macrophages in the presence of either control IgG or αvβ3 integrin blocking antibody (Ab). RT-PCR images are representative from two independent experiments. The ELISA values (mean ± standard deviation) are representative from two or three independent experiments (*n* = 4). *p ≤ 0.05 using a Student’s *t*-test. The densitometric quantification values for phospho-IκB (p-IκB) immunoblot represent the ratio of phospho-IκB:actin and the fold-induction was calculated after normalizing with the control 0 min group. The densitometric values represent the mean ± standard deviation from three independent studies. *p ≤ 0.05 using a Student’s *t*-test

As 25HC interacted with both α5β1 and αvβ3 integrin complexes and activated FAK (Figs. 1 and 2), we assayed whether these effects regulated the proinflammatory response triggered by 25HC. Inhibiting cell surface α5β1 integrin with integrin blocking antibody diminished TNF production from 25HC-treated primary mouse macrophages by 60% compared to control, IgG-treated cells (Fig. 5a). To validate this result, we silenced α5 integrin expression in THP-1 cells using small-interfering RNA (siRNA; Fig. 5b and Supplementary Fig. 5a). Compared with controls, these cells exhibited reduced expression of TNF (Fig. 5c) and IL-6 (Supplementary Fig. 5b) following 25HC treatment. Based on these results we predicted that lack of α5 integrin would abrogate NFκB activation by 25HC. To evaluate NFκB status, we used α5 integrin knockout (KO) human haploid (HAP1) cells^43^, generated by CRISPR-Cas9 technology (Supplementary Fig. 5c). Indeed, we observed reduced NFκB activation [i.e., diminished phospho-IκB status (Fig. 5d) and higher levels of IκB protein (Supplementary Fig. 5d)] in 25HC-treated α5 integrin KO HAP1 cells compared to wild-type (WT) HAP1 cells. Densitometric analyses of the phospho-IκB and IκB protein western blot data revealed significant induction of NFκB in 25HC-treated WT cells, but not in α5 integrin KO cells (Fig. 5d and Supplementary Fig. 5d).

To further confirm our result we next evaluated the proinflammatory response in 25HC-treated wild-type (WT) and β3 integrin-deficient (β3 integrin heterozygous β3^+/−^ mice) BMDMs. TNF production by β3 integrin-deficient BMDMs treated with 25HC was reduced by 62% compared to their WT counterparts (Fig. 5e). This result was further validated by treating human THP-1 macrophages with the human αvβ3 integrin blocking antibody LM609. LM609 reduced 25HC-induced production of TNF and IL-6 by 35% and 90%, respectively (Fig. 5f, g).

### 25HC-integrin-FAK amplifies proinflammatory response

**Fig. 6:**
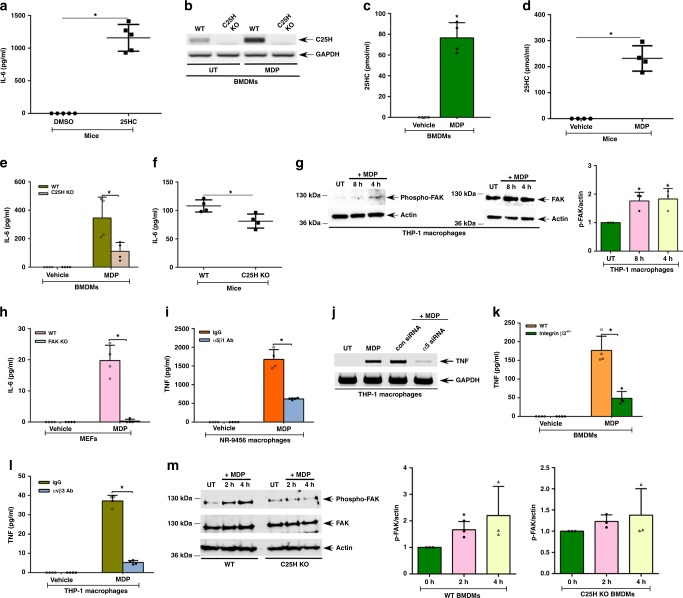
The 25HC-integrin-FAK signaling network is required for optimal Nod2 response. **a** IL-6 in serum of mice injected (via intraperitoneal route) with 25HC (50 mg/kg; 4 h) (*n* = 5). **b**, **c**, **e**, **g**–**m** Cells were treated with 25 µg/ml of MDP for 8 h and **d**, **f** mice were injected intraperitoneally with 20 mg/kg of MDP for 4 h. C25H expression, 25HC production, FAK activation, and proinflammatory response were assessed. **b** RT-PCR analyses of C25H expression in MDP-treated wild-type (WT) and C25H knockout (KO) BMDMs. **c** 25HC levels in the medium supernatant of MDP-treated BMDMs. **d** 25HC levels in the serum of WT mice treated with MDP (*n* = 4). **e** IL-6 secretion from WT and C25H KO BMDMs treated with MDP. **f** IL-6 levels in the serum of WT and C25H KO mice treated with MDP (*n* = 4). **g** Western blot and densitometric analyses of FAK activation (phospho-FAK, Tyr397) status in MDP-treated THP-1 macrophages. **h** IL-6 production from MDP-treated WT and FAK KO MEFs (mouse embryo fibroblasts). **i** IL-6 secretion from MDP-treated NR-9456 macrophages in the presence of either control IgG or α5β1 integrin blocking antibody (Ab). **j** RT-PCR analyses of TNF expression in MDP-treated THP-1 cells transfected with either control siRNA (con siRNA) or α5 integrin-specific siRNA (α5 siRNA). **k** TNF secretion from MDP-treated WT and β3 integrin-deficient (β3^+/−^ cells) BMDMs. **l** TNF secretion from MDP-treated THP-1 macrophages in the presence of either control IgG or αvβ3 integrin blocking antibody. **m** Western blot and densitometric analyses of FAK activation (phospho-FAK, Tyr925) status in MDP-treated WT and C25H KO BMDM. RT-PCR images are representative from two independent experiments. The ELISA values (mean ± standard deviation) are representative from two or three independent experiments (*n* = 4). *p ≤ 0.05 using a Student’s *t*-test. The densitometric quantification values for phospho-FAK (p-FAK) immunoblot represent the ratio of phospho-FAK:actin and the fold-induction was calculated after normalizing with the control untreated (UT) group. The densitometric values represent the mean ± standard deviation from three independent studies. *p ≤ 0.05 using a Student’s *t-*test

To examine whether extracellular 25HC can trigger proinflammatory response in mice, we administered (via intraperitoneal route) 25HC to C25H KO mice, as they do not produce any 25HC and the observed response will be entirely due to exogenously added 25HC. 25HC-treated mice exhibited a robust and systemic proinflammatory response as evidenced by detection of IL-6 in their serum (Fig. 6a). This suggested to us that the 25HC-integrin-FAK signaling network might regulate a proinflammatory response by facilitating the activation of PRRs, such as Nod2. Activation of Nod2 by muramyl dipeptide (MDP) led to a substantial increase in C25H expression and 25HC production by macrophages in vitro (Fig. 6b, c). MDP also triggered 25HC production in vivo (Fig. 6d). Furthermore, compared to WT cells, there was a significant inhibition in IL-6 production (reduced by 70%) in C25H KO BMDMs following MDP treatment (Fig. 6e). The specificity of 25HC during this process was evident as exogenous addition of 25HC to C25H KO cells restored a proinflammatory response following MDP administration (Supplementary Fig. 6a). The in vivo role of 25HC during a Nod2-mediated response was established as serum IL-6 levels in MDP-treated C25H KO mice were reduced 32% compared to WT mice (Fig. 6f).

As 25HC activated integrin-FAK signaling, we next assessed the role of the integrin-FAK pathway during Nod2 activation. MDP treatment resulted in an activation of FAK (i.e., detection of phospho-FAK) in macrophages (Fig. 6g). Densitometric analyses of the phospho-FAK western blot data revealed significant induction of FAK following MDP treatment (Fig. 6g). Furthermore, lack of FAK expression led to a complete loss of an MDP-induced proinflammatory response, as we detected negligible levels of IL-6 in FAK KO cells (Fig. 6h). Consistent with the latter result, inhibiting FAK activity in MDP-treated macrophages also resulted in complete abrogation in TNF production (Supplementary Fig. 6b). IL-6 production was also significantly compromised following inhibition of FAK activity in MDP-treated macrophages (Supplementary Fig. 6c). It should be noted that MDP treatment did not result in a loss of viability of either FAK KO macrophages or macrophages treated with a FAK inhibitor (Supplementary Table 1).

To assess the role of integrins, we evaluated TNF production from MDP-treated primary mouse macrophages in the presence of α5β1 integrin blocking antibody. There was a reduction of 65% in TNF production in the antibody-treated cells compared to the control (Fig. 6i). These results were further validated by using α5 integrin silenced macrophages (Fig. 5b and Supplementary Fig. 6d). Compared to control cells, α5 integrin-silenced cells exhibited reduced TNF and IL-6 expression following MDP treatment (Fig. 6j and Supplementary Fig. 6e). TNF and IL-6 production following MDP treatment of β3 integrin-deficient primary BMDMs was also reduced compared to controls by 75% and 70%, respectively (Fig. 6k and Supplementary Fig. 6f). This result was further validated in human macrophages. In cultures of human THP-1 macrophages treated with MDP, αvβ3 integrin blocking antibody incubation resulted in a dramatic reduction (reduced by 85%) of TNF production compared to control IgG-treated cells (Fig. 6l).

Finally, the role of 25HC in inducing integrin-FAK signaling during a Nod2-mediated innate response was established by evaluating FAK activation status in MDP-treated WT and C25H KO BMDMs. While MDP triggered FAK activation (i.e., detection of phospho-FAK) in WT cells, such activation was lacking in MDP-treated C25H KO cells (Fig. 6m). Densitometric analyses of the phospho-FAK western blot data revealed significant induction of FAK in MDP-treated WT cells, but not in C25H KO cells (Fig. 6m). These results demonstrated that 25HC promotes activation of integrin-FAK signaling, thereby amplifying proinflammatory response during a Nod2-mediated innate immune response.

### 25HC-integrin-FAK pathway regulates viral response

**Fig. 7:**
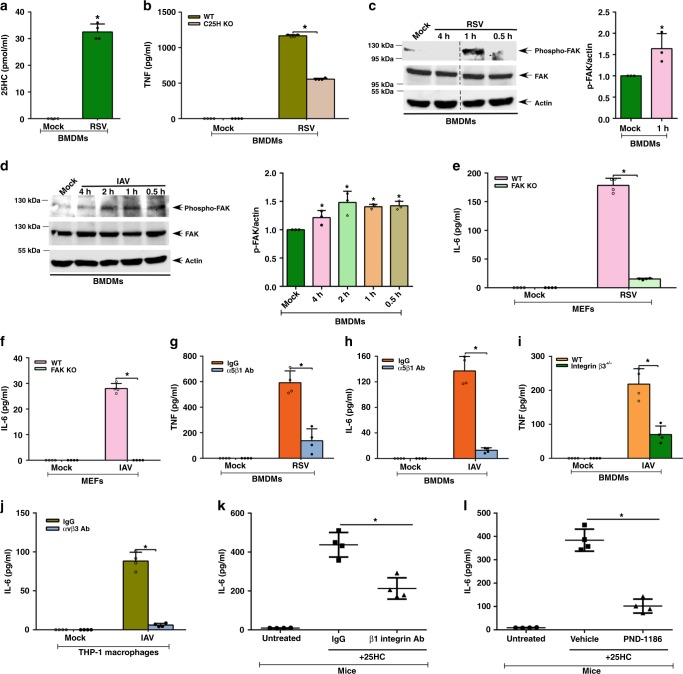
The 25HC-integrin-FAK signaling network regulates proinflammatory response during virus infection. **a** 25HC levels in the medium supernatant of BMDMs infected with human respiratory syncytial virus (RSV). **b** TNF production from wild-type (WT) and C25H knockout (KO) BMDMs infected with RSV. **c** Western blot and densitometric analyses of FAK activation (phospho-FAK, Tyr925) status in RSV-infected BMDMs. **d** Western blot and densitometric analyses of FAK activation (phospho-FAK, Tyr925) status in influenza A virus (IAV)-infected BMDMs. **e** IL-6 production from RSV-infected WT and FAK KO MEFs. **f** IL-6 production from IAV-infected WT and FAK KO MEFs. **g** TNF production from RSV-infected BMDMs in the presence of either control IgG or α5β1 integrin blocking antibody (Ab). **h** IL-6 production from IAV-infected BMDMs in the presence of either control IgG or α5β1 integrin blocking antibody. **i** TNF secretion from IAV-infected WT and β3 integrin-deficient (β3^+/−^ cells) BMDMs. **j** IL-6 secretion from IAV-infected THP-1 cells in the presence of either control IgG or αvβ3 integrin blocking antibody. **k** IL-6 in the lungs of mice injected (via intratracheal or I.T route) with 25HC (5 mg/kg; 6 h) in the presence of either IgG or β1 integrin blocking antibody (Ab) administered to the mice via I.T route (*n* = 4). **l** IL-6 in the lungs of mice injected (via I.T route) with 25HC (5 mg/kg; 6 h) in the presence of either vehicle (control) or FAK inhibitor (PND-1186) administered to the mice via I.T route (*n* = 4). The ELISA values (mean ± standard deviation) are representative from two or three independent experiments (*n* = 4). *p ≤ 0.05 using a Student’s *t*-test. The densitometric quantification values for phospho-FAK (p-FAK) immunoblot represent the ratio of phospho-FAK:actin and the fold-induction was calculated after normalizing with the control mock-infected group. The densitometric values represent the mean ± standard deviation from three independent studies. *p ≤ 0.05 using a Student’s *t*-test

Respiratory syncytial virus (RSV) and influenza A virus (IAV), two respiratory RNA viruses, activate Nod2 in macrophages, which, in turn, regulates a proinflammatory and innate immune response^14–17^. To assess whether 25HC-integrin-FAK signaling modulates a proinflammatory response during infection, we first assessed C25H expression and 25HC production in cultures of RSV-infected macrophages. RSV induced C25H expression (Supplementary Fig. 7a), which resulted in 25HC production (Fig. 7a). IAV also triggered 25HC production (Supplementary Fig. 7b). The importance of 25HC during RSV infection was demonstrated by the dampening of the proinflammatory response in RSV-infected C25H KO macrophages (Fig. 7b). Specifically, TNF and IL-6 production were reduced by 52% and 44%, respectively, following RSV infection of C25H KO macrophages (Fig. 7b and Supplementary Fig. 7c). In accord with a previous report^26^, we also observed a diminished proinflammatory response following IAV infection of C25H KO BMDMs (Supplementary Fig. 7d). Thus, 25HC amplified the proinflammatory response following RSV and IAV infection.

Next, we examined the role of the integrin-FAK pathway during infection. Both IAV and RSV infection induced FAK activation in macrophages as phospho-FAK was detected in infected cells following western blot analyses (Fig. 7c, d). Densitometric analyses of the phospho-FAK western blot data revealed significant induction of FAK following infection (Fig. 7c, d). A role for FAK in regulating the proinflammatory response to RSV infection is apparent from the drastic reduction in IL-6 release from RSV-infected FAK KO cells (Fig. 7e). Similarly, TNF and IL-6 production was diminished in RSV-infected macrophages treated with a FAK inhibitor (Supplementary Fig. 7e, f). The loss of a proinflammatory response was also observed in IAV-infected FAK KO cells (Fig. 7f) and in macrophages treated with a FAK inhibitor (Supplementary Fig. 7g). Neither treatment condition nor infection status altered cell viability in these studies (Supplementary Table 1). Moreover, our results are not due to FAK regulating infectivity, as RSV and IAV viral infection was similar in WT and FAK KO cells (Supplementary Fig. 8a, b).

Next, we evaluated the role of α5β1 and αvβ3 integrins during virus infection. TNF production of RSV-infected cells declined by 80% following incubation with α5β1 integrin blocking antibodies (Fig. 7g). Likewise, incubation of IAV-infected cells with α5β1 integrin blocking antibody resulted in a 90% reduction in IL-6 production (Fig. 7h). Furthermore, TNF production was reduced by 70% in β3 integrin-deficient macrophages (BMDMs from β3^+/−^ mice) following IAV infection compared to controls (Fig. 7i). This result was further validated in IAV-infected THP-1 human macrophages as anti-human αvβ3 integrin blocking antibodies diminished IL-6 production in these cells by 95% (Fig. 7j).

It has been suggested that C25H possesses antiviral functions against various viruses^44^. A recent study showed that C25H lacks in vivo antiviral activity against IAV^26^. In accord with this result, we did not observe any difference in RSV and IAV infectivity in WT versus C25H KO BMDMs (Supplementary Fig. 8c, d). Taken together, the results of our study indicated an involvement of 25HC-integrin-FAK signaling network in positively regulating proinflammatory response during RSV and IAV infection.

In order to demonstrate that 25HC confers a proinflammatory response via integrin-FAK pathway in an in vivo setting, we next used a physiologically relevant mouse model. Gold et al.^26^ demonstrated that IAV infection triggers induction of C25H. This result was confirmed as we detected 25HC in the lungs of IAV-infected mice (Supplementary Fig. 7h). In addition, studies performed by Gold et al.^26^ revealed that the absence of 25HC in C25H KO mice results in diminished proinflammatory response in IAV-infected mice^26^. To investigate the role of the integrin-FAK pathway in 25HC-mediated activation of a proinflammatory response in a pathophysiological setting, mimicking IAV infection, we administered 25HC to the mouse airway either in the presence or absence of a β1 integrin blocking antibody, previously employed to inhibit integrin activation in the mouse airway^45^, or the FAK inhibitor (PND-1186), which has been used in mice to block FAK activation^46^. This experimental design in a physiological setting is ideal to directly assess whether 25HC utilizes the integrin-FAK pathway to induce proinflammatory response in vivo. 25HC-dependent proinflammatory response in the lung was significantly diminished following administration of β1 integrin blocking antibody (Fig. 7k) and FAK inhibitor (Fig. 7l). In summary, our results indicated that the integrin-FAK pathway plays an important physiological role in triggering 25HC-mediated response.

## Discussion

The ability of integrins, clustered within matrix adhesion sites, to regulate signal transduction pathways that not only modulate cell adhesion and motility but also gene expression has been the subject of numerous studies^1–6,9–12,27,28,47^. Integrin-mediated signaling requires activation of the integrin molecule, a process which involves conformational changes resulting in enhanced ligand binding. Outside-in integrin signaling requires integrin interaction and activation by an extracellular ligand. This results in conformational changes in the cytoplasmic domains of the integrin heterodimer. The new conformation facilitates the recruitment and/or activation of signaling intermediates that bind to the cytoplasmic tails.

Apart from binding to ECM ligands, integrins can also interact with other non-conventional extracellular ligands such as adhesion molecules (e.g., ICAM, VCAM), phospholipase A2, and fractalkine^35,36,48^. These ligands appear to act as activators promoting (‘‘priming’’) ligand interaction to induce the necessary conformational change in the cytoplasmic tail to trigger signaling^1–3,5^.

To date non-protein ligands for integrins have not been described. As discussed above, only proteinaceous extracellular ligands for integrin have been described and a non-protein ligand for integrin has not been identified. Thus, the detection of a lipid as an integrin ligand is surprising. Moreover, we show that the oxysterol lipid 25HC directly interacts with α5β1 and αvβ3 integrins to trigger integrin activation. Furthermore, our modeling analyses suggest that 25HC, like fractalkine and sPLA2-IIA, interacts with αvβ3 integrin via site-II in the extracellular domain of the β3 integrin subunit^35,36^. Our modeling also indicates that this interaction affects the conformation of the specificity-determining loop (SDL), located between residues Lys156-Gly189 of the βI domain of β3 integrin. SDL determines binding to site I of numerous RGD motif-containing ligands, including fibrinogen, von Willebrand factor, vitronectin, and fibronectin^32,38,39,49,50^. We propose that 25HC binds to site-II, located in the globular heads of inactive αvβ3 integrin, when the latter is in a bent conformation. This event could then “prime” activation of integrin signaling, a possibility supported by our observation that active αvβ3 integrin is found in the podosomes of THP-1 cells only after 25HC treatment and 25HC promotes activation of αvβ3 integrin in FAs of A549 cells. However, in an alternative indirect mechanism, 25HC binding to site-II could induce conformational changes in the SDL, which may promote efficient “high-affinity” binding of RGD containing ligands (e.g., fibronectin, laminin etc.) in the site I ligand-binding pocket. Furthermore, such conformational change in SDL may also dictate “specificity” in terms of selective binding of RGD ligand to site I. Thus, we envision that both direct and indirect mechanisms of integrin activation by 25HC may either occur simultaneously or are temporally distinct. Furthermore, two mechanisms of integrin activation by 25HC may be utilized to maximize integrin signaling strength.

The data we have presented here regarding 25HC is an example of a lipid regulating integrin activation via direct binding. There was a single report in 1992 that an unsaturated acid or an isoprenoid acid acts as an activator of leukocyte integrins^51^. However, neither the nature of the lipid involved, nor its mechanism of action, were elucidated by the authors of the study^51^. Regardless, the ability of 25HC to modulate integrin activation adds to its already extensive list of activities of 25HC in modulating various cellular responses^26,44,52–55^.

As part of our efforts to elucidate the biological and functional roles of 25HC-mediated integrin binding, we have uncovered a previously unknown mechanism that connects innate immunity to integrin signaling. During an innate immune response, the PRR Nod2 senses unique pathogen-derived PAMPs and, as a consequence, induces NFκB and MAPK activation and a subsequent release of proinflammatory mediators.

**Fig. 8:**
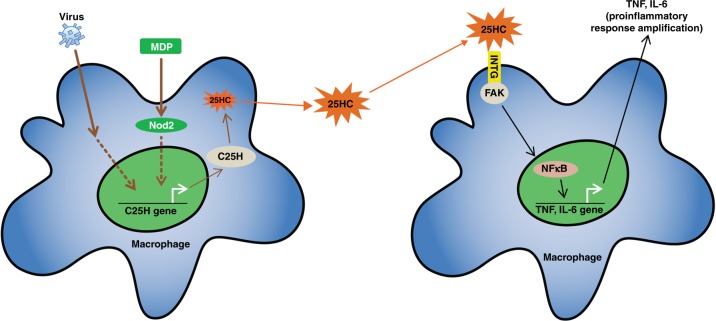
A schematic model showing regulation of proinflammatory response by 25HC-integrin-FAK signaling network. Nod2 activation and virus (RSV and IAV) infection triggers expression of C25H, which results in production of 25HC. Extracellular 25HC activates integrin-FAK-NFκB signaling. Thus, 25HC links PRR (Nod2) pathway with integrin-FAK-NFκB signaling to confer optimal proinflammatory response following Nod2 activation and virus infection. INTG, integrin; FAK, focal adhesion kinase

Based on our study, we propose a “biphasic” model for Nod2 activation during viral infection (Fig. 8). We suggest that two “discrete”, but “mutually inclusive” signals are required for optimal proinflammatory response following Nod2 activation by PAMPs. First, a signal (first phase) occurring in “primed” cells promotes production of the first wave of proinflammatory mediators. PRR activation in these cells results in NFκB and MAPK activation and subsequent release of sub-optimal levels of proinflammatory mediators. During this process, C25H is also induced in “primed” cells. 25HC generated due to enzymatic activity of C25H is released to the extracellular milieu. Next, a signal (second phase) triggers production of a second wave of proinflammatory mediators. This is executed by the paracrine/autocrine action of extracellular 25HC. In the case of paracrine action, 25HC activates integrin-FAK signaling in “bystander” cells (i.e., the cells lacking activated PRR) to trigger proinflammatory response. We postulate that existence of two signals for triggering optimal PRR response during infection is an essential component of a host’s “regulatory” mechanism to maintain proper “checks and balances” during inflammation. Thus, our studies have unfolded a role of the PRR-25HC-integrin-FAK-NFκB signaling network in amplifying inflammatory response.

In summary, our studies have led to identification of 25HC as an extracellular integrin “lipid” ligand involved in activation of integrin-FAK signaling to regulate innate immune response.

## Methods

### Viruses and cells

Influenza A [A/*PR*/8/34 (H1N1)] virus (IAV) and human respiratory syncytial virus (RSV A2 strain) were purified by centrifuging two times on discontinuous sucrose gradients^14,56^. Bone marrow-derived macrophages (BMDMs) were obtained from femurs and tibias of wild type (WT), C25H knockout (KO), NFκB p105 KO, and integrin β3^+/−^ mice. BMDMs were cultured for 6–8 days and plated for experiments in 1640 RPMI, 10% FBS, 100 IU/ml Penicillin, 100 µg/ml Streptomycin (Gibco, Maryland, USA), and 20 ng/ml GM-CSF (PeproTech, New Jersey, USA). NR-9456 macrophages (immortalized BMDMs) (a gift from Michael T. Berton, UT Health Science Center at San Antonio; Bei Resources, Virginia, USA; catalog no. NR-9456), RAW 264.7 macrophages (ATCC, Virginia, USA; catalog no. TIB-71), mouse embryo fibroblasts (WT and FAK KO MEFs) (ATCC; catalog no. CRL-2645 and CRL-2644) were maintained in complete DMEM containing 10% FBS, 100 IU/ml Penicillin, 100 µg/ml Streptomycin (Gibco). A549 cells (human lung epithelial cells) (ATCC; catalog no. CCL-185) were maintained in complete MEM containing 10% FBS, 100 IU/ml Penicillin, 100 µg/ml Streptomycin and 4 mM l-glutamine. The human monocyte cell line (THP-1) (ATCC; catalog no. TIB-202) were cultured in 1640 RPMI, 10% FBS, 100 IU/ml Penicillin, 100 µg/ml Streptomycin, 1 mM sodium pyruvate, 10 mM HEPES, and 50 µM β-mercaptoethanol (Sigma Aldrich, Missouri, USA). THP-1 cells were differentiated by treatment with 100 nM phorbol 12-myristate 13-acetate (PMA) (Sigma Aldrich). WT (catalog no. C631) and α5 integrin null (catalog no. HZGHC001085c001) human haploid (HAP1) cells, created using CRISPR-Cas9 technology were purchased from Horizon Discovery Inc (Vienna, Austria). HAP1 cells were maintained in IMDM, 10% FBS, 100 IU/ml Penicillin, 100 µg/ml Streptomycin (Gibco). All cell lines used in the current study were tested for *Mycoplasma* using Universal *Mycoplasma* Detection kit (ATCC).

### Cell treatment and infection

Cells were treated with freshly prepared 25HC (50 µM, 8 h) (Steraloids, Rhode Island, USA) and the Nod2 activator muramyl dipeptide (MDP) (25 µg/ml, 8 h) (InvivoGen, California, USA). In some experiments, cells were pre-treated with either FAK inhibitor (5 µM) (PF-431396; Sigma Aldrich) or NFκB inhibitor (10 µM) (Bay-11–7082; InvivoGen) for 1 h or 30 min, respectively. Subsequently, these cells were either treated with various agents (25HC and MDP) or infected with viruses (RSV and IAV). Cells were infected with purified IAV or RSV at the multiplicity of infection (MOI) of 1. Virus adsorption was performed for 1.5 h (at 37 °C) in serum-free, antibiotic-free OPTI-MEM medium (Gibco). Following adsorption, cells were washed twice with PBS and infection was continued for an additional 8 h or 16 h in the presence of serum containing complete medium with or without treatment. For in vitro experiments (i.e., experiments with cultured cells), standard biological replicates of *n* = 4 were used.

### Mice

Wild type (WT) (stock no. 000664), C25H knockout (KO) (stock no. 016263), NFκB p105 KO (stock no. 006097), and β3 integrin-deficient (β3 integrin heterozygous β3^+/−^ mice) (stock no. 004669) mice were purchased from the Jackson Laboratory (Maine, USA). All the mice used in the study were female 6–8-week-old C57BL/6J mice. WT and C25H KO mice were injected intraperitoneally with MDP (20 mg/kg). C25H KO mice were also injected intraperitoneally with 25HC (50 mg/kg). At 4 h post treatment, serum was collected from treated mice. In order to study the role of the integrin-FAK pathway in promoting 25HC-mediated proinflammatory response in mice, we analyzed IL-6 levels in mouse lungs after 25HC administration to the airway in the presence of either β1 integrin blocking antibody (anti-mouse CD29, clone-9EG7; BD Pharmigen, California, USA) or FAK inhibitor PND-1186 (MedKoo Biosciences, North Carolina, USA). Female 6–8-week-old C57BL/6J mice were injected intratracheally with either IgG or β1 integrin blocking antibody (20 μg/ml). At 2 h post treatment, mice were injected intratracheally with either vehicle control (ethanol) or 25HC (5 mg/kg). At 6 h post 25HC treatment, lungs were isolated. IL-6 levels in the lung homogenate were analyzed by ELISA. In a separate experiment, female 6–8-week-old C57BL/6J mice were injected intratracheally with either vehicle control (water) or FAK inhibitor PND-1186 (50 mg/kg). At 1 h post treatment, mice were injected intratracheally with either vehicle control (ethanol) or 25HC (5 mg/kg). At 6 h post 25HC treatment, lungs were isolated. IL-6 levels in the lung homogenate was analyzed by ELISA. In some experiment, mice were infected with influenza A virus (IAV). Female 6–8-week-old C57BL/6J mice were intratracheally inoculated with either medium (vehicle control) or IAV (1 × 10^4 ^pfu/mouse). At 2 days post infection, lungs were isolated. 25HC levels in the lung homogenate was analyzed by 25HC detection kit. Animal experiments were approved and carried out in accordance with the guidelines established by the Institutional Animal Care and Use Committee (IACUC) of Washington State University.

### Integrin blocking

Cell surface α5β1 and αvβ3 integrin were inhibited in vitro by pre-treating cells with α5β1 integrin (75 μg/ml, MAB 2514) and αvβ3 integrin (10 μg/ml, LM609) blocking antibody (Millipore, Massachusetts, USA), respectively. IgG served as a control for the experiments. Following pre-treatment, cells were either infected with viruses (RSV and IAV) or treated with 25HC and MDP. For in vivo experiments, mice were treated with either IgG (control) or β1 integrin blocking antibody (anti-mouse CD29, clone-9EG7).

### Silencing with siRNA

Differentiated human THP-1 macrophages were transfected with either 100 pmol of control siRNA or human α5 integrin siRNA using Lipofectamine 2000 (Invitrogen, California, USA). At 16 h post transfection, cells were treated with either MDP or 25HC. Control siRNA and human α5 integrin siRNA were purchased from Santa Cruz Biotechnology (Texas, USA).

### Western blotting

FAK activation was assessed by performing western blotting with phospho-FAK (mouse Tyr925; catalog no. 3284 and human Tyr397; catalog no. 8556) (1:500) and FAK (1:1000) antibodies (catalog no. 13009) (Cell Signaling, Massachusetts, USA). Western blotting with IκB (catalog no. 4812) (1:1000) and phospho-IκB (catalog no. 2859) (1:1000) antibodies (Cell Signaling) was performed to examine NFκB activation status. As indicated, α5 integrin (catalog no. ab150361) (1:500) and αv integrin (catalog no. ab179475) (1:1000) antibodies (Abcam, Cambridge, UK) were also used for western blot analyses. The actin antibody (catalog no. A300-485A) (1:5000) was purchased from Bethyl Laboratories (Texas, USA). In some experiments, protein bands from the western blots were quantified by using ChemiDoc™ XRS + software Image Lab 5.1 (BioRad). Uncropped western blots are shown in Supplementary Fig. 10.

### Cytokine and 25HC detection assay

TNF and IL-6 levels in the medium supernatant and mice serum were assessed by using a specific ELISA kit (eBioscience, California, USA). 25HC levels in the medium supernatant and mice serum were measured with a 25HC detection kit (MyBiosource, California, USA). Please note that for the macrophages, the ELISA values of the experimental group (i.e., 25HC, MDP-treated cells or RSV, IAV-infected cells) represent values obtained following subtraction of background signal measured in the control group (i.e., vehicle-treated cells or mock-infected cells). The background values for the MEF control group were below the detection limit of the ELISA kit.

### Lactate dehydrogenase cytotoxicity assay

LDH-Cytotoxicity Assay kit-II (BioVision, California, USA) was used to evaluate cell viability. Briefly, medium supernatant was incubated with LDH Reaction Mix. The absorbance at 450 nm was then measured using a micro-plate reader. Percent cytotoxicity was calculated according to the manufacturer’s instructions. As a positive control, cells were treated with a supplier provided agent that induces cellular toxicity.

### Biotinylation of 25HC

The EZ-link® TFPA-PEG3-Biotin kit (Thermo Fisher Scientific, Massachusetts, USA) was used to biotinylate 25HC. This kit is appropriate for biotinylating non-protein macromolecules like lipids. As DMSO was the vehicle for 25HC we also treated an equal volume of DMSO to serve as a control. Please note that we do not expect DMSO to be biotinylated as it does not possess the necessary side-groups for the biotinylation reaction. Biotinylation was performed in the dark as per the manufacturer’s instructions. To assess successful biotinylation of 25HC, biotin-25HC was pulled down with NeutrAvidin–agarose beads (Thermo Fisher Scientific) and avidin bound material was eluted by incubating the beads with 10 mM EDTA and 95% formamide at a pH of 8.2 for 15 min. Eluted material was assessed for 25HC using a 25HC detection kit. In addition to 25HC, 27-hydroxycholesterol (27HC) (Steraloids), and 4β-hydroxycholesterol (4βHC) (Steraloids) were also biotinylated.

### Interaction of biotinylated 25HC with integrins

Macrophages (BMDMs) were pre-cooled to 4 °C for 1 h, then incubated at 4 °C with either biotin-25HC (50 µM) or DMSO control for 4 h. Cells were lysed with 1% TritonX-100 (Sigma) and protease inhibitor (Roche, Basel, Switzerland) in PBS. Cell lysates were incubated with NeutrAvidin-agarose beads (Thermo Fisher Scientific) (12 h at 4 °C). After exhaustively washing the agarose beads with wash buffer (10 mM Tris-HCL and protease inhibitor in PBS), the proteins bound to avidin–agarose beads were subjected to western blotting with integrin antibodies. This experiment was repeated using biotin-25HC, biotin-27HC, and biotin-4βHC.

Biotin-25HC was also used to assess direct binding of 25HC to purified integrin proteins. Biotin-25HC was incubated (12 h at 4 °C) with avidin–agarose beads. These complexes were then further incubated (12 h at 4 °C) with purified α5β1 integrin or αvβ3 integrin, which were purchased from Yo proteins AB (Huddinge, Sweden). After exhaustively washing the agarose beads, the avidin–agarose bound proteins were subjected to western blotting with α5 and αv integrin antibodies.

### Interaction of tritiated ^3^H-25HC with integrins

^3^H-25HC was purchased from PerkinElmer (Massachusetts, USA). Protein G agarose beads were incubated (12 h, 4 °C) with control IgG or antibodies against α5 or αv integrin. Antibody-conjugated beads were then incubated (12 h, 4 °C) with purified α5β1 and αvβ3 integrin proteins (Yo proteins AB, Huddinge, Sweden). The antibody–antigen bound beads were washed and incubated with ^3^H-25HC (0.01 µCi) for 12 h at 4 °C. Following incubation, the beads were exhaustively washed and bound radioactivity was counted using a TRI-CARB 2900TR liquid scintillation counter.

### Reverse transcription-PCR (RT-PCR)

Total RNA was extracted using TRIzol reagent (Life Technologies, California, USA) following the manufacturer’s instructions. MultiScribe reverse transcriptase (Applied Biosystem, California, USA) was used to synthesize template cDNA. PCR was performed using Apex^®^ 2X Taq Red master mix (Genesee Scientific, California, USA) in a final reaction volume of 25 μl. The amplified PCR products were visualized in a 1–2% agarose gel. Amplified glyceraldehyde-3-phosphate dehydrogenase (GAPDH) gene PCR product was used as a loading control. PCR primers for each gene are listed in Supplementary Table 3.

### Immunofluorescence microscopy

Cell preparations were processed for laser scan confocal immunofluorescence microscopy^57^. Briefly, A549 and THP-1 cells grown on coverslips were incubated with DMSO (vehicle control) or 0.5 µM 25HC for 2 h. They were then fixed by incubation with 3.7% formaldehyde for 5 min and extracted with 0.5% TritonX-100 for 7 min. Primary antibodies, diluted 1:100, were incubated in a solution of 0.05% tween-20 and 5% normal goat serum (Jackson ImmunoResearch Laboratories, Pennsylvania, USA) in PBS for 1 h at 37 °C. The mouse monoclonal antibody against αvβ3 integrin (MAB1976Z, clone LM609) were purchased from Millipore. The mouse monoclonal antibody against active β3 integrin (EBW107, clone AP5) was purchased from Kerafast (Massachusetts, USA). The rabbit monoclonal antibody against paxillin (ab32084, clone Y113) was purchased from Abcam. Secondary antibodies, diluted 1:200, were incubated in 0.05% tween-20 in PBS for 1 h at 37 °C. The fluorescein-conjugated goat anti-mouse IgG (115-095-166) and rhodamine-conjugated goat anti-rabbit IgG (111-025-144) antibodies were purchased from Jackson ImmunoResearch Laboratories. Samples were imaged at room temperature using a TCS SP5 confocal microscope equipped with a 63 × 1.4 NA objective and version 2.4.1 (build 6384) of the LASAF imaging suite (Leica Microsystems, Illinois, USA). Analyses of focal adhesions and podosomes were performed by counting the number present per cell when treated with DMSO or 0.5 µM 25HC for 2 h. Such experiments were repeated three times and followed by one-sided Wilcoxon rank-sum tests to compare conditions.

Analyses of focal adhesions were performed as described by us^58^ and others^59^. Analyses of focal adhesions were performed by counting all distinct fluorescently labeled integrin clusters that localized to the basal portion of cell surface when viewed via confocal microscopy following a 2 h treatment with DMSO or 0.5 µM 25HC. Using ImageJ and constant parameters for each experiment, background was subtracted, local contrast was enhanced by using CLAHE^60^, and thresholding was performed. Contiguous blocks of thresholded pixels were grouped together to define focal adhesions using the R package Bioi^61^.

### Cell motility assays

Cells were imaged every 5 min for 2 h using a DMi8 conventional fluorescent microscope (Leica Microsystems) equipped with a 5 × 0.12 NA objective, a 37 °C heated chamber (Pathology Devices), and a DFC365 FX FCAM2 camera. Images were acquired using the LAS X image acquisition suite (version 1.0.0.12269). Cell positions were tracked using image cross-correlation velocimetry as implemented in MetaMorph software version 7.8.0.0 (Molecular Devices, California, USA). Cell positions were used to calculate speed and processivity. The latter was defined as the maximum displacement from a cell’s origin divided by the length of the path it traveled.

### Integrin internalization assay

αvβ3 integrin internalization was assayed using established protocols^62^. Briefly, 24 h after plating onto glass coverslips, A549 cells were treated for 30 min on ice with antibodies against αvβ3 integrin (1:100 in culture medium, MAB1976Z, Millipore) and then again with fluorescein-conjugated goat anti-mouse antibodies (1:200 in culture medium, 115-095-166, Jackson ImmunoResearch Laboratories). This was followed by a 30 min incubation at 37 °C in culture medium supplemented with DMSO or 0.5 µM 25HC. Finally, cells were incubated for 30 min on ice with rhodamine-conjugated goat anti-mouse antibodies (1:200 in culture medium, 115-025-166, Jackson ImmunoResearch Laboratories). Samples were imaged using a TCS SP5 confocal microscope (Leica Microsystems). Internalization was quantified by calculating Spearman’s correlation between the two fluorescent channels for all pixels exceeding the background fluorescence level in the fluorescein channel.

### Surface plasmon resonance studies

To study biomolecular interaction between integrin and 25HC in real-time, SPR was performed using a Biacore 3000 instrument (GE Healthcare, Piscataway, New Jersey) according to the manufacturer’s instructions. Purified human αvβ3 integrin protein (Yo proteins AB, Huddinge, Sweden) was covalently immobilized on a flow cell of CM5 chip (carboxy-methylated dextran coated) in 10 mM sodium acetate buffer, pH 4.0, using EDC/NHS amine coupling chemistry at 25 °C. The unused dextran surface was then inactivated by injecting 1 M Ethanolamine, pH 8.5. Similarly, the blank control flow cell was simultaneously activated and inactivated without the protein for background subtraction of any non-specific response. For kinetic analyses, increasing concentrations of 25HC (0 nM, 16 nM, 40 nM, 160 nM, 640 nM, 1.6 µM) in the running buffer HBS-P (10 mM HEPES, 0.15 M NaCl, 0.005% polysorbate 20, pH 7.4) with 1% DMSO were injected at a flow rate of 20 μl/min for 150 s. Following dissociation, the chip surface was regenerated with the running buffer. The background subtracted SPR sensorgrams were quantitatively evaluated to determine *K*_D_ (apparent affinity constant) by using the Biacore 3000 Evaluation Software (GE Healthcare) and the Langmuir 1:1 binding model.

### Competitive binding analyses

A modified version of “ELISA-type” binding assay^34–36^ was used to study binding of 25HC to human αvβ3 integrin protein in the absence and presence of the competitor comprising of purified chemokine domain (CD) of recombinant human fractalkine protein (Peprotech, New Jersey, USA). For this assay, 96-well Nunc micro-well plate (Thermo Fisher Scientific) was coated with purified human αvβ3 integrin protein (200 ng of protein/well in PBS containing 1 mM MnCl_2_)^35,63^. Following incubation (16 h, 4 °C), the wells were washed with PBS/0.1% BSA and subsequently blocked with PBS/2% BSA for 3 h (at room temperature). Blocked wells were washed with PBS/0.1% BSA and then either vehicle (water) or purified fractalkine-CD protein (1000 ng/well in HEPES-Tyrodes buffer) was added to the wells. Following 2 h incubation (at room temperature), the wells were washed with PBS/0.1% BSA, and subsequently ^3^H-25HC (in PBS/0.5% BSA) was added to the wells. Following 16 h incubation (at 4 °C), the wells were washed with PBS/0.1% BSA and the bound radioactivity (representing ^3^H-25HC bound to αvβ3 integrin in the absence and presence of fractalkine-CD protein) was counted using a TRI-CARB 2900TR liquid scintillation counter.

### Molecular modeling studies

The three-dimensional (3D) structure of the ligand molecule 25HC was built in Molecular Operating Environment (MOE) (Chemical Computing Group, Montreal, Canada) and subsequently geometry optimized and Mulliken charge calculations were performed in Gaussian16 program (Gaussian, Wallingford, Connecticut, USA) using the DFT/B3LYP method and the 6–31G** basis set.

Molecular docking and dynamics studies were conducted using the published crystal structures of extracellular domains of integrins αvβ3 (PDB code 1L5G and 3IJE) in complex with ARG-GLY-ASP (RGD) ligand^64^ and unbound states, respectively. The crystal structure was prepared using “Protein Preparation” module in MOE (missing atoms, residues, and H atoms were added, protonation states of residues were assigned using protonate 3D within the MOE). The crystal structure (PDB code 3IJE) represents the complete unconstrained ectodomain^65^ and short C-terminal transmembrane stretches of the αv and β3 subunits.

Molecular docking of 25HC at the classical ‘RGD’’-binding site (site I), as well as site-II was performed in MOE. The ligand molecule was docked using guided-docking mode in which the ‘RGD’’ cyclic peptide bound to αvβ3^64^ was used as a template to place 25HC within the binding site. For site-II positioning, the integrin-binding site was defined by selected residues (Supplementary Table 2).

Two placement methods namely Alpha PMI, and Alpha Triangle and Proxy Triangle were employed to generate best-docked poses of 25HC. In the Alpha PMI method, poses are generated by aligning ligand conformations’ principal moments of inertia to a randomly generated subset of alpha spheres in the receptor site. Whereas in the Alpha Triangle and Proxy triangle method, poses are generated by superposition of the ligand atom triplets and triplets of receptor site points representing locations of tight packing. The protein–ligand complex structures generated by the placement method were further refined by induced fitting of protein side chains while the backbone atoms were held fixed. The generated poses were scored using the enthalpy-based “Affinity dG” scoring function, followed by the “London dG” scoring function^66^ at the refinement stage.

All MD simulations were performed using the AMBER16 package^67^ under isothermal/isobaric (NPT) ensemble with periodic boundary conditions. The AMBER ff14SB force field, General AMBER force field (GAFF)^68^, and TIP3P^69^ water model were employed for the protein, including bivalent and monovalent ions, 25HC, and solvent molecules, respectively. We used the Leap program from Antechamber tools (AmberTools14)^70^ to generate the parameter/topology and input coordinate files. The net charge of the integrin–25HC complex was kept neutral by adding 34 Na^+^ ions at positions of high-negative electron potential around the protein–ligand complex. The system was immersed in a truncated octahedral box of pre-equilibrated TIP3P water molecules in such a way that no atoms in the protein–ligand complex were closer than 8 Å to any of the sides of the solvent box. The counter ions and solvent molecules were briefly minimized (1000 steps of steepest descent minimization followed by another 1000 steps of conjugate gradient method) to remove any bad contacts with the complexes. The rest of the system was position-restrained using a force constant of 100 kcal/(mol × *Å*^2^). This step was followed by another 2000 step minimization of the whole solvated complex. The readjustment of solvent molecules to the potential field of the protein–ligand complex was achieved by subsequent heating and equilibration steps. The system was heated to a final temperature of 300 K in three steps: 0 to 100 K for 10 ps (NVT), 100 to 200 K for 20 ps, and 200 to 300 K for another 20 ps, all under NPT conditions (1 atm pressure). Isotropic position scaling was used to maintain the pressure, and a relaxation time of 2 ps was used. During the heating phases, the protein–ligand complexes were position-restrained with a force constant of 10 kcal/(mol × *Å*^2^). Langevian dynamics was used in all stages to control the temperature using a collision frequency of 1.0 ps^−1^. The final solvent equilibration step was performed for 50 ps under NPT conditions as above. The SHAKE algorithm was used to constrain bonds involving hydrogen, allowing a time step of 2 fs. Lennard-Jones and electrostatic interactions were calculated explicitly within a cutoff of 1.2 nm, and long range electrostatic interactions were calculated by particle mesh Ewald summation. The production simulation was carried out for 200 ns and the trajectory file was written for every 20,000 steps resulting in 20,000 frames.

The unbound and bound forms of integrin–25HC complexes from MD simulations were analyzed for its conformational change and structural integrity of both α- and β-subunits. The secondary structures and overall integrity of the interacting domains were found to be stable throughout the simulation period. Principal component analyses (PCA) was performed to characterize the cumulative and overall motion of the αvβ3 integrin, resulting from the atomic fluctuations due to 25HC binding. The PCA analyses was performed using ProDy^71^ interface in NMWiz (Normal Model Wizard) plugin implemented in VMD. In PCA analyses, the first principal component typically consists of the largest root-mean-square fluctuation (RMSF). The correlation matrices were calculated from the mass-adjusted Cartesian coordinates representing ensemble average of all snapshots from the production phase (200 ns) simulation. We have restricted the analyses to only Cα atoms, as they are less perturbed by statistical noise and provide significant characterization of the essential space motions. Comparative analyses of both bound and unbound species was performed by calculating the cross-correlation matrices, using Carma 1.7 program^72^ to investigate the dynamic cross-correlated displacements of Cα atoms of αvβ3 in the MD simulation trajectories. To visualize the direction and extent of the principal motions of αvβ3 in its bound state, porcupine plot analyses was performed using the PorcupinePlot.tcl script in VMD.

### Statistical approach

ELISA data were analyzed using Graphpad Prism software (6.0) and significance test was carried out using Student’s *t*-test. Densitometric values of the western blots were quantified by using ChemiDoc™ XRS + software Image Lab 5.1 (BioRad) and significance test was carried out using Student’s *t*-test. Immunocytochemistry and cell motility data were analyzed using one-way and two-way Wilcoxon rank-sum tests.

### Reporting Summary

Further information on experimental design is available in the Nature Research Reporting Summary linked to this article.

## Supplementary information

Supplementary Information

Reporting Summary

Description of Additional Supplementary Files

Supplementary Movie 1

Supplementary Movie 2

Supplementary Movie 3

Supplementary Movie 4

Source Data

## Data Availability

The data supporting the findings are available within the article and Supplementary Information. The source data of Figs 1c–f, 2e–i, 4b, c, 5a, e–g, 6a, c–f, h, i, k, l, 7a, b, e–l, and Supplementary Figs. 1a, b, 2a, b, d, 4a–c, 6a–c, f, 7b–h are provided as a Source Data file. All other data are available from the authors upon reasonable request.
