# Supplementary material for: Integrin activation by the lipid molecule 25-hydroxycholesterol induces a proinflammatory response
Source: Nat Commun. 2019 Apr 1;10:1482. doi: 10.1038/s41467-019-09453-x (PMC6443809; doi:10.1038/s41467-019-09453-x)
Supplement: Supplementary file 1 — Supplementary Information [file 41467_2019_9453_MOESM1_ESM.pdf]

Integrin activation by the lipid molecule 25-hydroxycholesterol induces a proinflammatory response

Pokharel et al.

Supplementary Information

Supplementary information contains Supplementary Figures 1-10, Supplementary Tables 1-3, Supplementary Notes, and Supplementary references

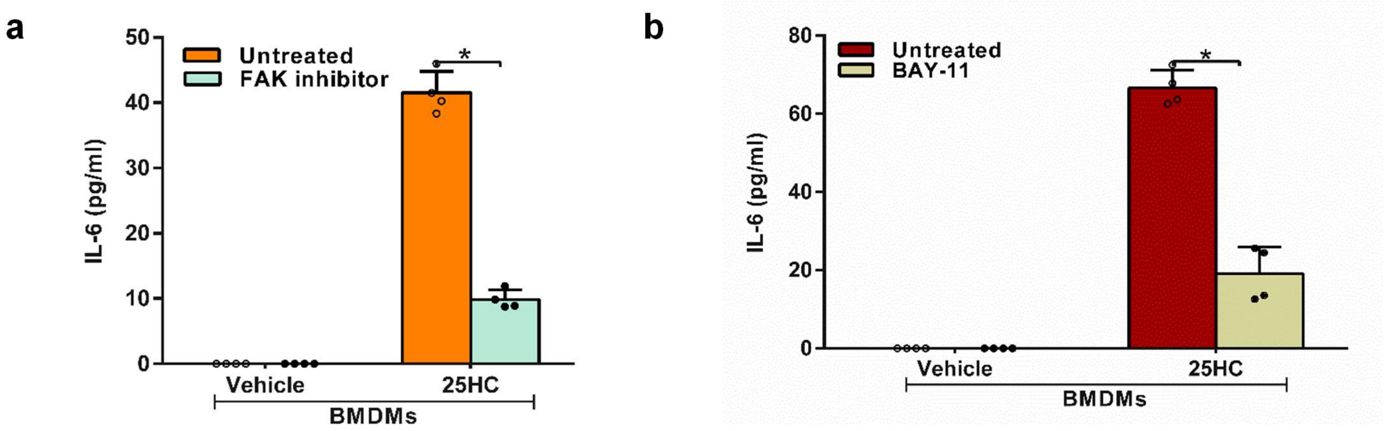

Supplementary Figure 1. The FAK-NFκB signaling pathway mediates a 25HC-dependent proinflammatory response. **a** IL-6 secretion from BMDMs treated with 25HC in the absence or presence of FAK inhibitor (5 μM). **b** IL-6 secretion from BMDMs treated with 25HC in the absence or presence of NFκB inhibitor Bay 11-7082 (BAY-11) (10 μM). The ELISA values (mean ± standard deviation) are representative from two or three independent experiments (n=4). *p ≤ 0.05 using a Student's t test.

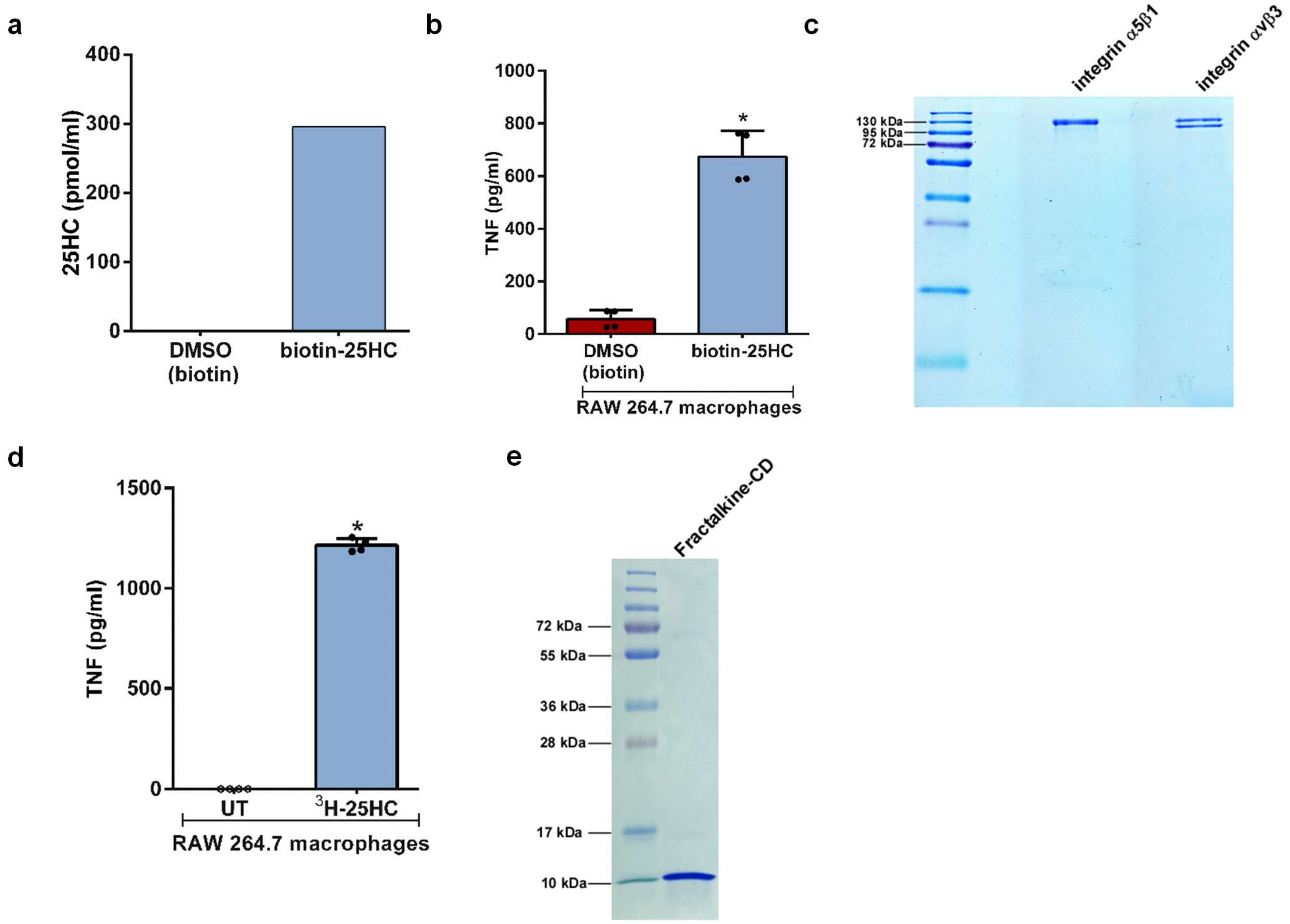

Supplementary Figure 2. Analyses of the functions of biotinylated 25HC. Biotinylation reaction was performed in the presence of either 25HC or DMSO (control). **a** To examine conjugation of biotin to 25HC, biotinylated 25HC (biotin-25HC) was precipitated with avidin-agarose. Beads were washed and bound material was eluted. The eluted material was assayed for 25HC by using 25HC detection kit. In this experiment DMSO (vehicle control was subjected to biotinylation reaction) served as a control. **b** TNF production from RAW 264.7 macrophages treated with either DMSO (control) or biotin-25HC for 8h. **c** SDS-PAGE analyses of purified human α5β1 and αvβ3 integrin proteins. The gel was stained with Coomassie Blue. **d** TNF secretion from untreated (UT) and tritiated ^{3}H-25HC treated (8h) RAW 264.7 macrophages. **e** SDS-PAGE analyses of purified recombinant human fractalkine protein (comprising of the chemokine domain or CD of fractalkine protein; molecular weight of ~ 9 kDa). The gel was stained with Coomassie Blue. The ELISA values represent the mean ± standard deviation. *$p \leq 0.05$ using a Student's t test.

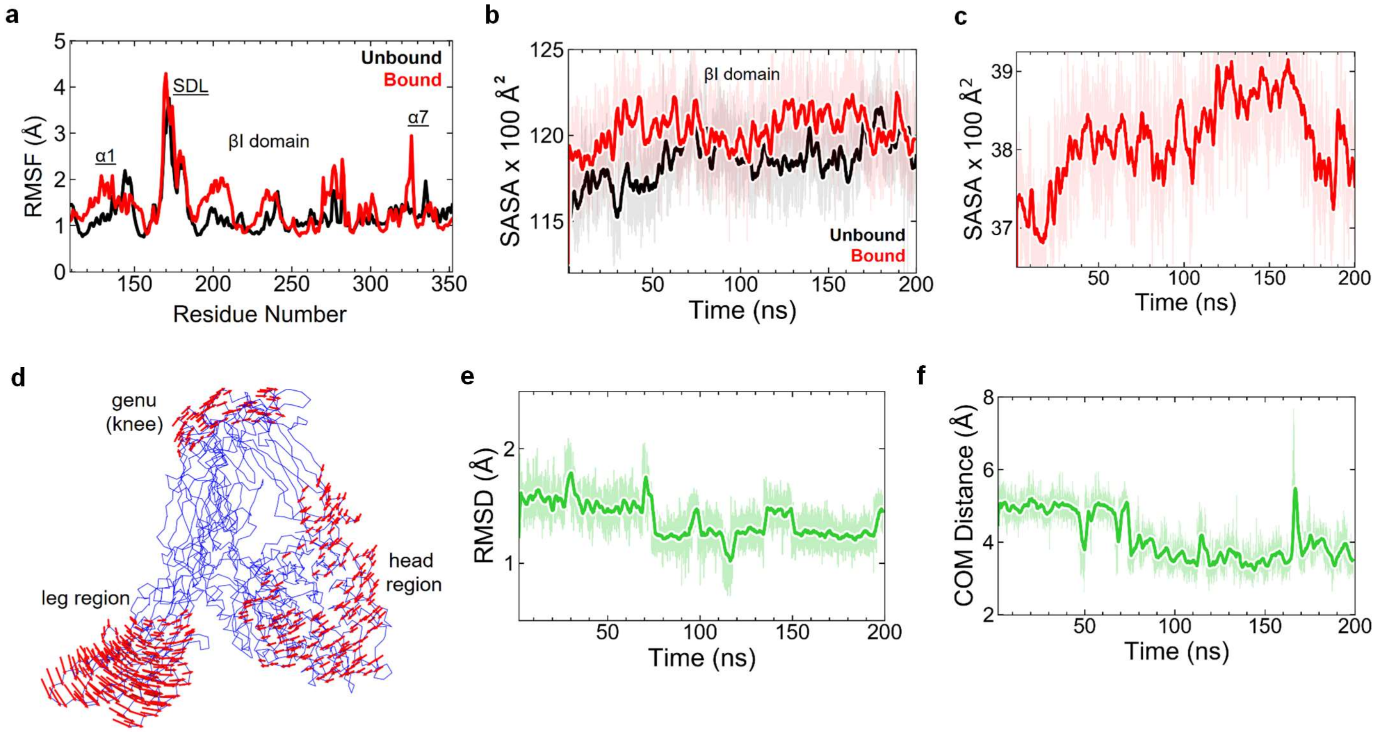

Supplementary Figure 3. 25HC affects the conformational dynamics of αvβ3 integrin. a) Root-mean-square-fluctuations (RMSF) of residues from the βI domain: in addition to significant conformational changes at α-1 helix, the specificity-determining loop (SDL) undergoes notable conformational change leading to opening of the crevice between the β-propeller and βI domains, possibly changing site I to a high-affinity binding site for RGD-containing ligands. **b)** Conformational changes following 25HC binding resulted in significant change in the solvent accessible surface area (SASA) of the βI domain compared to that of unbound αvβ3 integrin. **c)** The increase in SASA of residues from SDL and the β-propeller loop due to the conformational change, upon 25HC binding. **d)** Porcupine plot reveals the overall dynamic motion of αvβ3 integrin head and genu (knee) regions with respect to the leg region, upon 25HC binding. **e)** Root-mean-square-deviation (RMSD) of 25HC within the binding site during the entire 200 ns simulation time. **f)** Distance between center of mass (COM) of all binding site residues and that of 25HC atoms, indicating stable van der Waal (steric) interactions between these two molecules throughout the 200 ns simulation time.

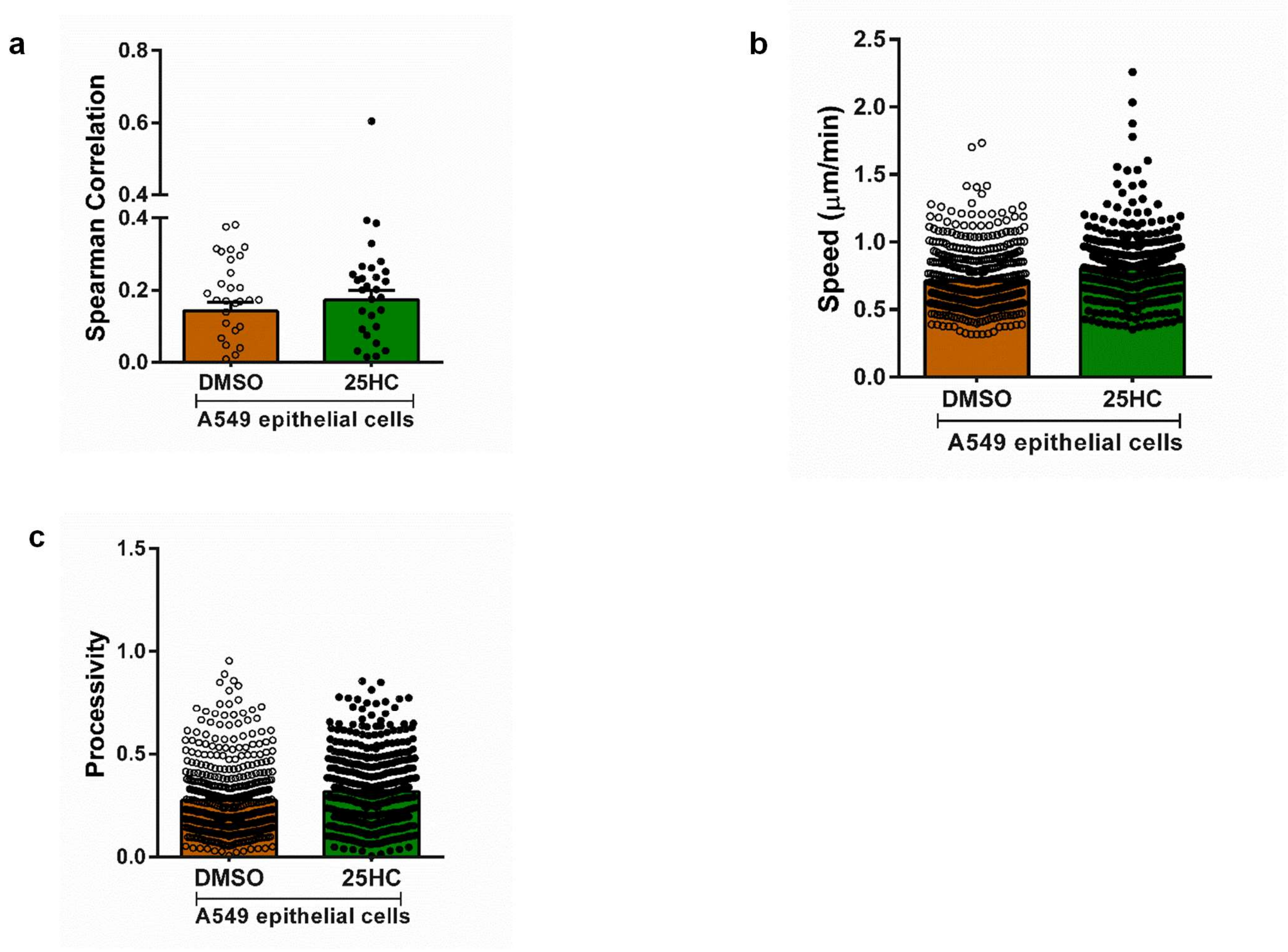

Supplementary Figure 4. 25HC does not regulate cell motility and integrin internalization. **(a)** To quantify internalization, A549 cells were treated for 30 min on ice with antibodies targeting αvβ3 integrin then again with fluorescein-conjugated secondary antibodies. Integrins were allowed to internalize at 37^0C for 30 min before treatment with rhodamine-conjugated secondary antibodies. Cells were imaged using a TCS SP5 confocal microscope and the Spearman correlation between the two fluorophores was calculated (3 experiments, > 12 cells per experiment; n ≥30). Time-lapse microscopy was performed for 2 h with a 5 min imaging interval on DMSO (control) or 25HC (0.5 μM) treated A549 cells (3 experiments, > 100 cells per experiment; n ≥ 300). No major changes to a cell speed (**b**) or processivity (a measure of directed migration) (**c**) were observed. The values represent mean ±SEM.

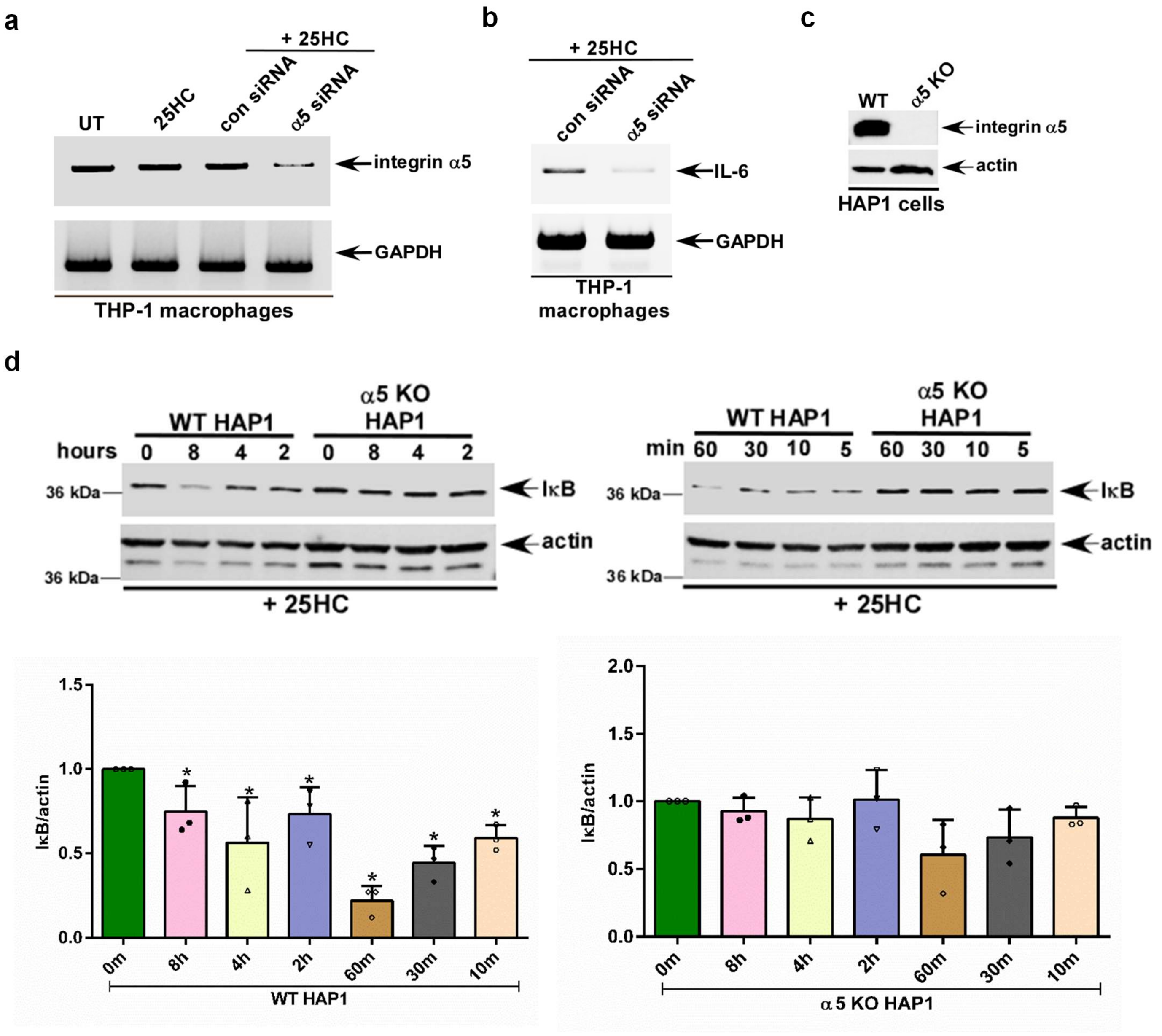

Supplementary Figure 5. α5 integrin regulates 25HC-mediated NFκB activation and proinflammatory response. **a** RT-PCR analyses of α5 integrin mRNA expression in untreated (UT) and 25HC treated THP-1 cells transfected with either control siRNA (con siRNA) or α5 integrin specific siRNA (α5 siRNA). **b** RT-PCR analyses of IL-6 expression in 25HC treated THP-1 cells transfected with either control siRNA or α5 siRNA. **c** Western blotting analyses of α5 integrin protein expression in wild type (WT) and α5 integrin knockout (KO) HAP1 cells. **d** Western blot and densitometric analyses of IκB protein status in 25HC treated WT and α5 integrin KO HAP1 cells. The densitometric quantification values for IκB protein immunoblot represent the ratio of IκB:actin and the fold-induction was calculated after normalizing with the control 0h group. The densitometric values represent the mean ± standard deviation from three independent Western blots. *$p \leq 0.05$ using a Student's t test.

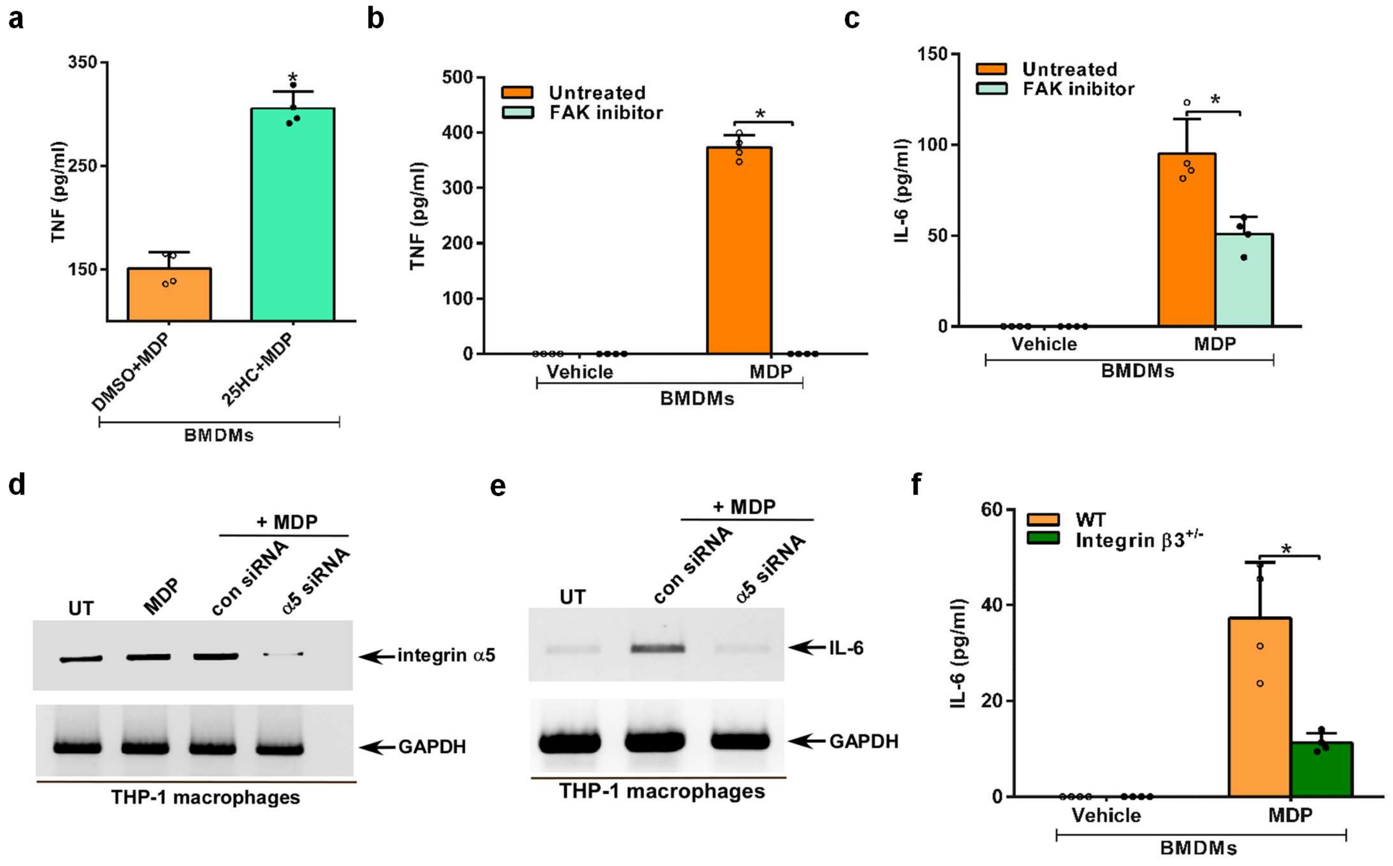

Supplementary Figure 6. Nod2 response is regulated by the 25HC-integrin-FAK signaling network. **a** TNF secretion from C25H KO cells treated with MDP in presence of DMSO (control) or 25HC. **b, c** TNF and IL-6 secretion from BMDMs treated with MDP in the absence or presence of FAK inhibitor. **d** RT-PCR analyses of α5 integrin mRNA expression in untreated (UT) and MDP treated THP-1 cells transfected with either control siRNA (con siRNA) or α5 integrin specific siRNA (α5 siRNA). **e** RT-PCR analyses of IL-6 expression in MDP treated THP-1 cells transfected with either control siRNA or α5 siRNA. **f** IL-6 secretion from MDP treated WT and β3 integrin deficient ($\beta3^{+/-}$ cells) BMDMs. RT-PCR images are representative from two independent experiments. The ELISA values (mean ± standard deviation) are representative from two or three independent experiments (n=4). *$p \leq 0.05$ using a Student's t test.

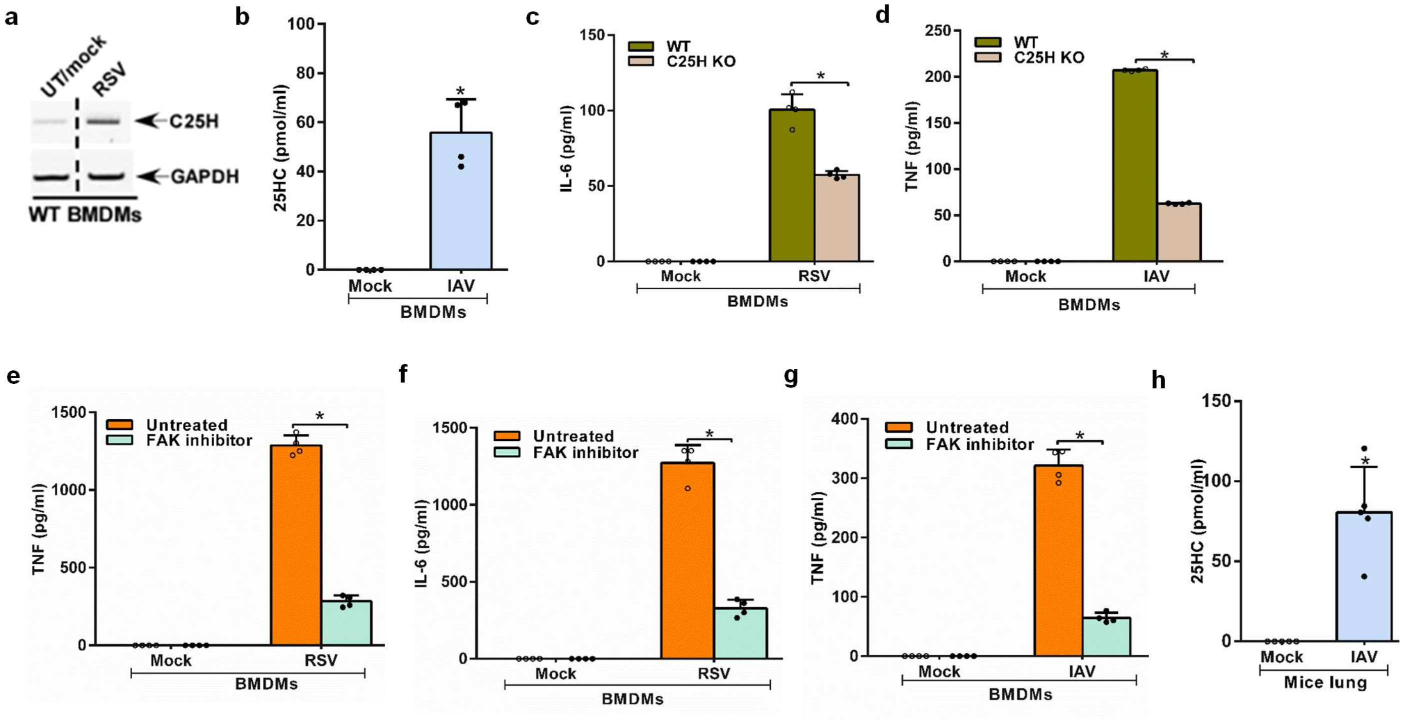

Supplementary Figure 7. The 25HC-integrin-FAK signaling network regulates the proinflammatory response during virus infection. **a** RT-PCR analyses of C25H expression in RSV infected BMDMs. **b** 25HC secretion from IAV infected BMDMs. **c** IL-6 secretion from RSV infected wild type (WT) and C25H knockout (KO) BMDMs. **d** TNF secretion from IAV infected WT and C25H KO BMDMs. **e, f** TNF and IL-6 secretion from RSV infected BMDMs in presence or absence of the FAK inhibitor. **g** TNF secretion from IAV infected BMDMs in presence or absence of the FAK inhibitor. **h** Levels of 25HC in the lung of IAV infected mice (n=5). The ELISA values (mean ± standard deviation) are representative from two or three independent experiments (n=4). *$p \leq 0.05$ using a Student's t test.

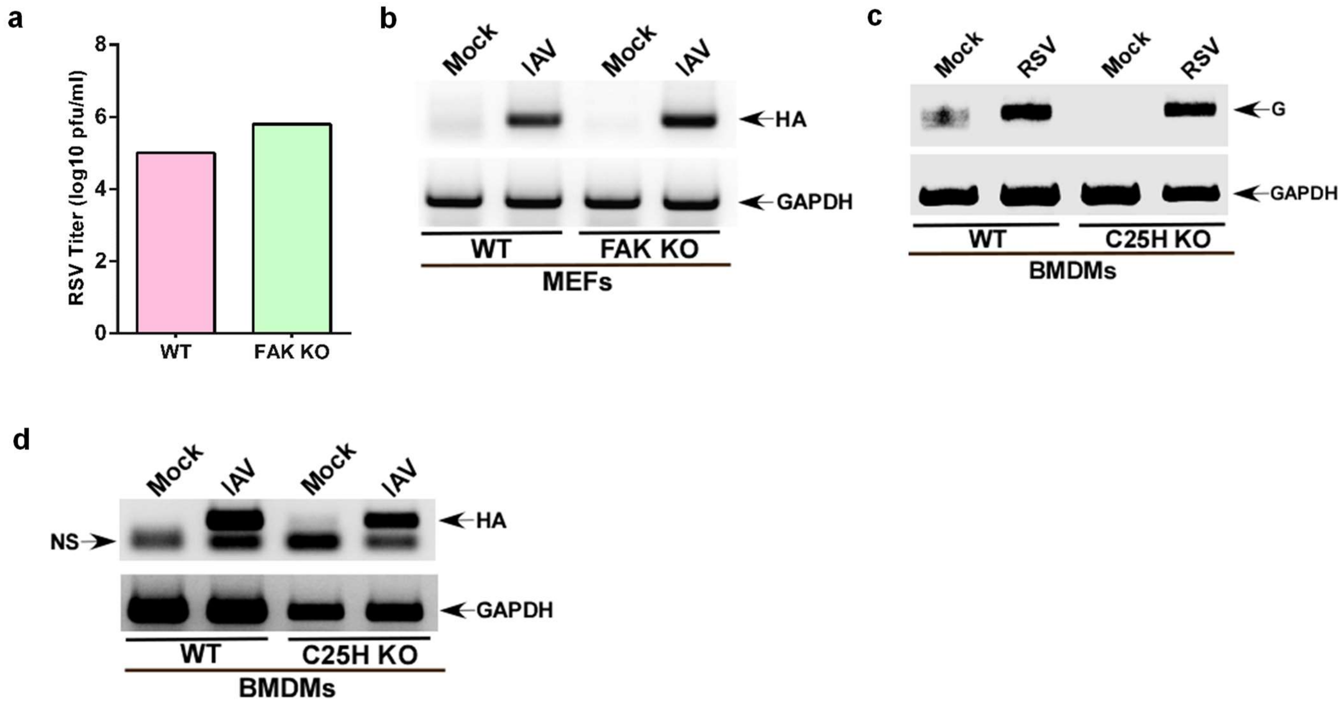

Supplementary Figure 8. The 25HC-integrin-FAK signaling network does not regulate virus infectivity. **a** RSV titer in wild type (WT) and FAK knockout (KO) mouse embryo fibroblasts (MEFs) following 8h infection at 1 MOI. **b** IAV hemagglutinin (HA) expression in WT and FAK KO MEFs following 16h infection at 1 MOI. **c** RSV glycoprotein (G) expression in WT and C25H KO BMDMs following 8h infection at 1 MOI. **d** IAV hemagglutinin (HA) expression in WT and C25H KO BMDMs following 16h infection at 1 MOI.

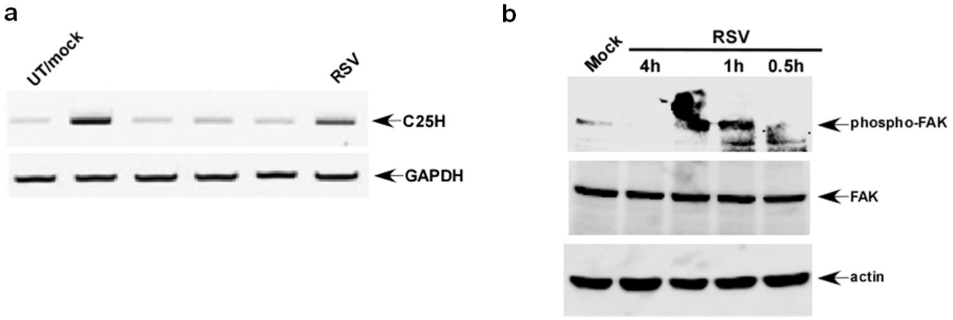

Supplementary Figure 9. **Unprocessed blot**. **a** Unprocessed blot corresponding to supplementary figure. 7a. **b** Unprocessed blot corresponding to figure. 7c.

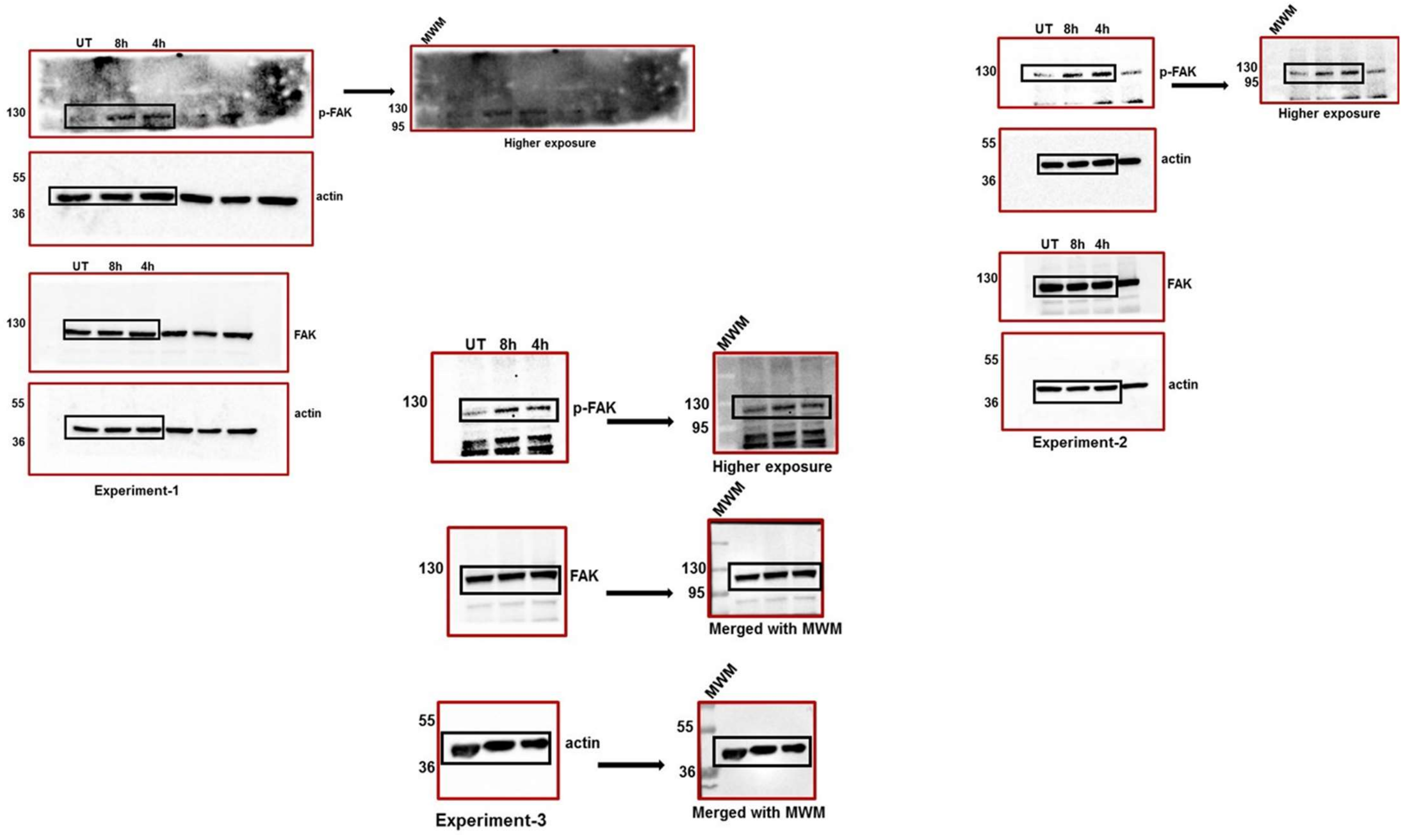

Supplementary Figure 10. Uncropped Western blots corresponding to the figures in the manuscript.

Supplementary Figure 10a. Western blots corresponding to Figure. 1b. The blot shown in experiment-1is displayed in Figure. 1b. Blots shown in experiment-2 and experiment-3 are independent repeats of the same experiment. Red and black outlines indicates uncropped and cropped images, respectively. For this experiment, after transfer, the membrane was cut into strips corresponding to the size of p-FAK, FAK and actin. The strips were then incubated with corresponding antibodies. Densitometric analyses shown in Fig. 1b was performed based on the quantification of the protein bands shown in experiment-1, experiment-2 and experiment-3. In some blots higher exposure is shown so that molecular weight marker (MWM) impression is visible. In addition, some blots were merged with MWM.

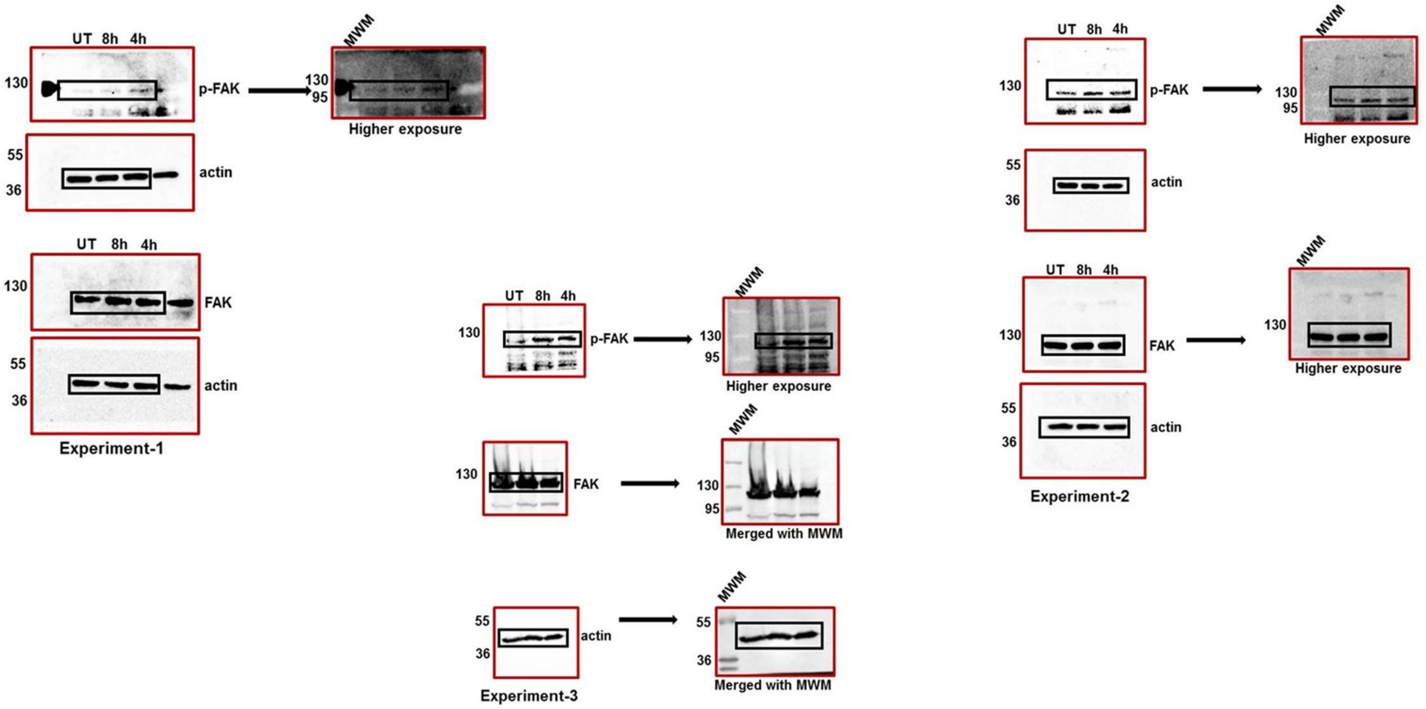

Supplementary Figure 10. **Uncropped Western blots corresponding to the figures in the manuscript.**

Supplementary Figure 10b. Western blots corresponding to Figure. 6g. The blot shown in experiment-1is displayed in Figure. 6g. Blots shown in experiment-2 and experiment-3 are independent repeats of the same experiment. Red and black outlines indicates uncropped and cropped images, respectively. For this experiment, after transfer, the membrane was cut into strips corresponding to the size of p-FAK, FAK and actin. The strips were then incubated with corresponding antibodies. Densitometric analyses shown in Fig. 6g was performed based on the quantification of the protein bands shown in experiment-1, experiment-2 and experiment-3. In some blots higher exposure is shown so that molecular weight marker (MWM) impression is visible. In addition, some blots were merged with MWM.

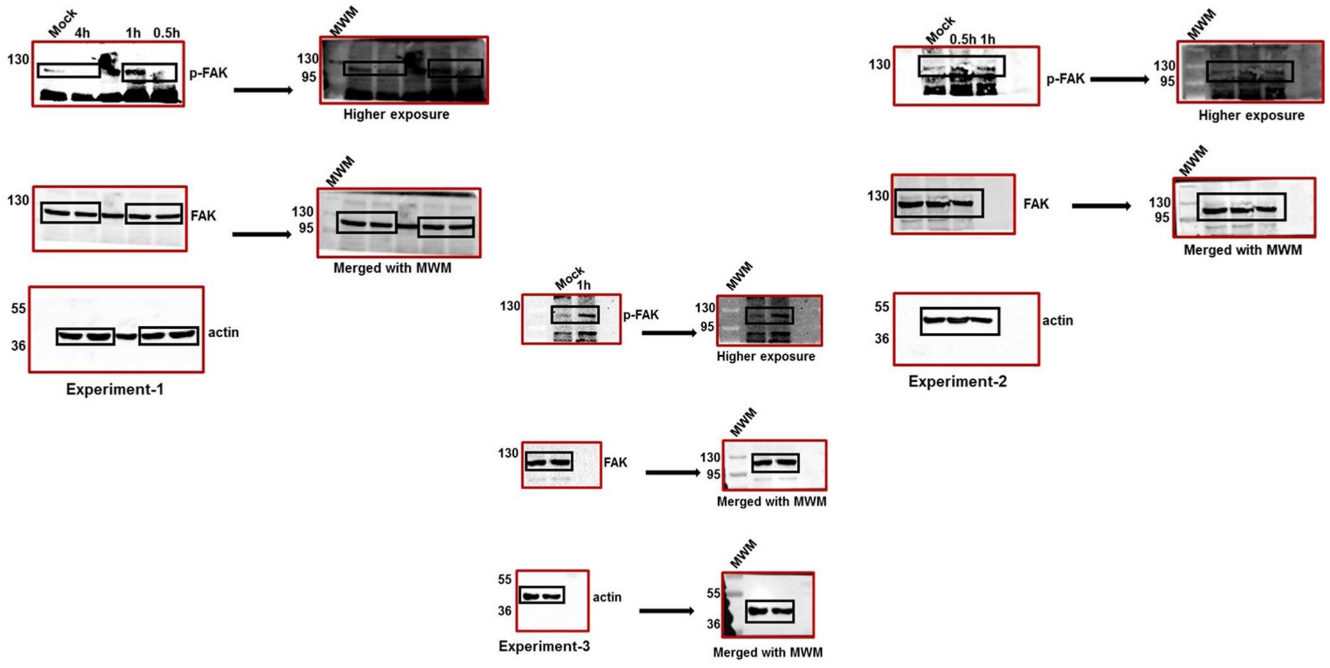

Supplementary Figure 10. Uncropped Western blots corresponding to the figures in the manuscript.

Supplementary Figure 10c. Western blots corresponding to Figure. 7c. The blot shown in experiment-1is displayed in Figure. 7c. Blots shown in experiment-2 and experiment-3 are independent repeats of the same experiment. Red and black outlines indicates uncropped and cropped images, respectively. For this experiment, after transfer, the membrane was cut into strips corresponding to the size of p-FAK, FAK and actin. The strips were then incubated with corresponding antibodies. Densitometric analyses shown in Fig. 7c was performed based on the quantification of the protein bands shown in experiment-1, experiment-2 and experiment-3. In some blots higher exposure is shown so that molecular weight marker (MWM) impression is visible. In addition, some blots were merged with MWM.

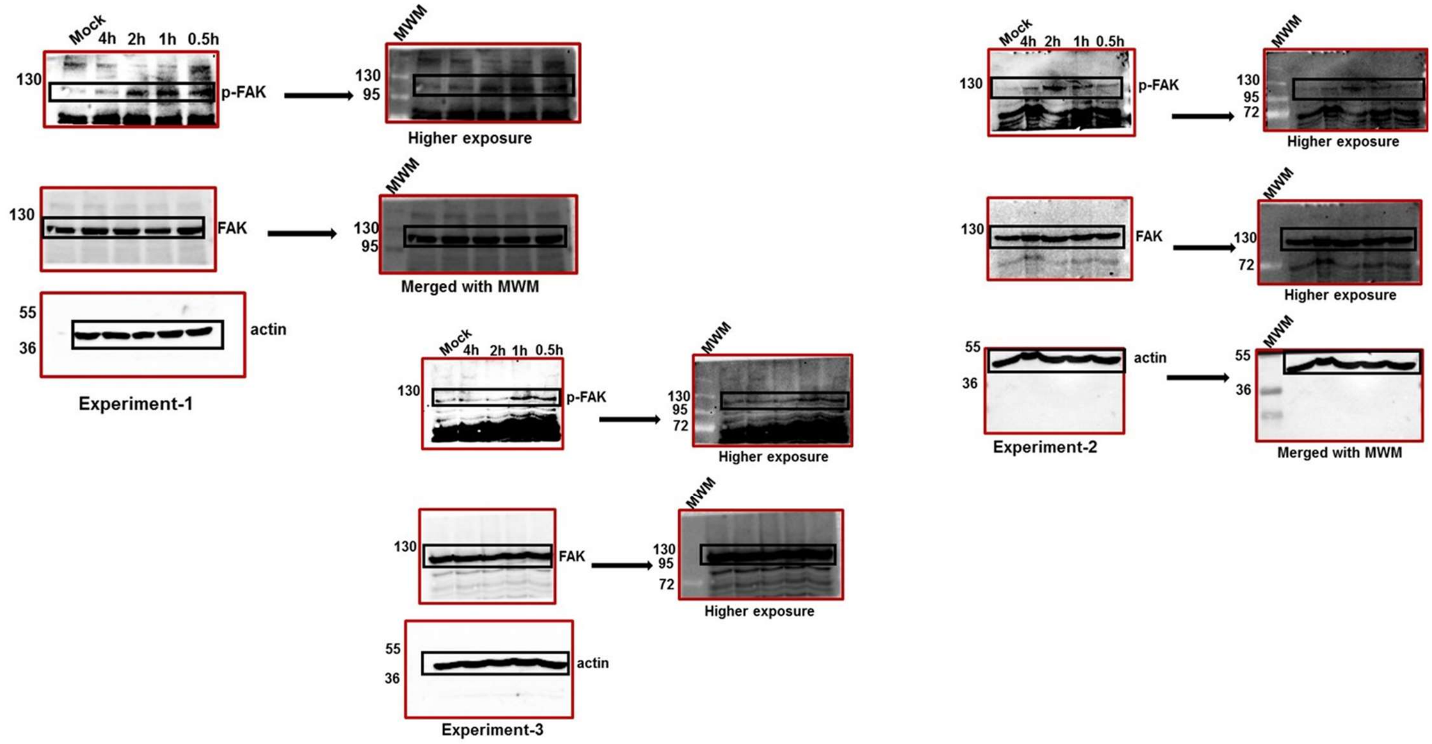

Supplementary Figure 10. **Uncropped Western blots corresponding to the figures in the manuscript.**

Supplementary Figure 10d. Western blots corresponding to Figure. 7d. The blot shown in experiment-1is displayed in Figure. 7d. Blots shown in experiment-2 and experiment-3 are independent repeats of the same experiment. Red and black outlines indicates uncropped and cropped images, respectively. For this experiment, after transfer, the membrane was cut into strips corresponding to the size of p-FAK, FAK and actin. The strips were then incubated with corresponding antibodies. Densitometric analyses shown in Fig. 7d was performed based on the quantification of the protein bands shown in experiment-1, experiment-2 and experiment-3. In some blots higher exposure is shown so that molecular weight marker (MWM) impression is visible. In addition, some blots were merged with MWM. In some blots higher exposure is shown so that molecular weight marker (MWM) impression is visible. In addition, some blots were merged with MWM.

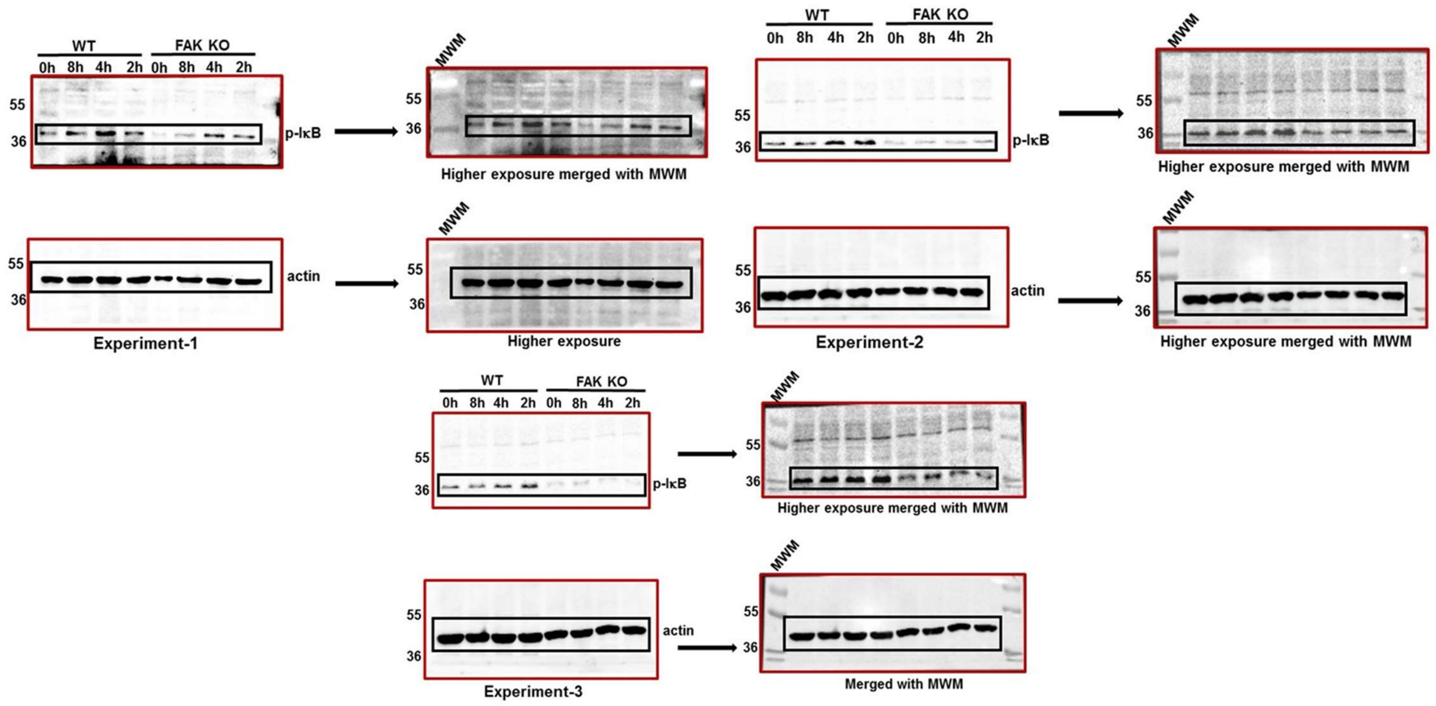

Supplementary Figure 10. **Uncropped Western blots corresponding to the figures in the manuscript.**

Supplementary Figure 10e. Western blots corresponding to Figure. 1g. The blot shown in experiment-1is displayed in Figure. 1g. Blots shown in experiment-2 and experiment-3 are independent repeats of the same experiment. Red and black outlines indicates uncropped and cropped images, respectively. For this experiment, after transfer, the membrane was cut into strips corresponding to the size of p-IκB and actin. The strips were then incubated with corresponding antibodies. Densitometric analyses shown in Fig. 1g was performed based on the quantification of the protein bands shown in experiment-1, experiment-2 and experiment-3. In some blots higher exposure is shown so that molecular weight marker (MWM) impression is visible. In addition, some blots were merged with MWM.

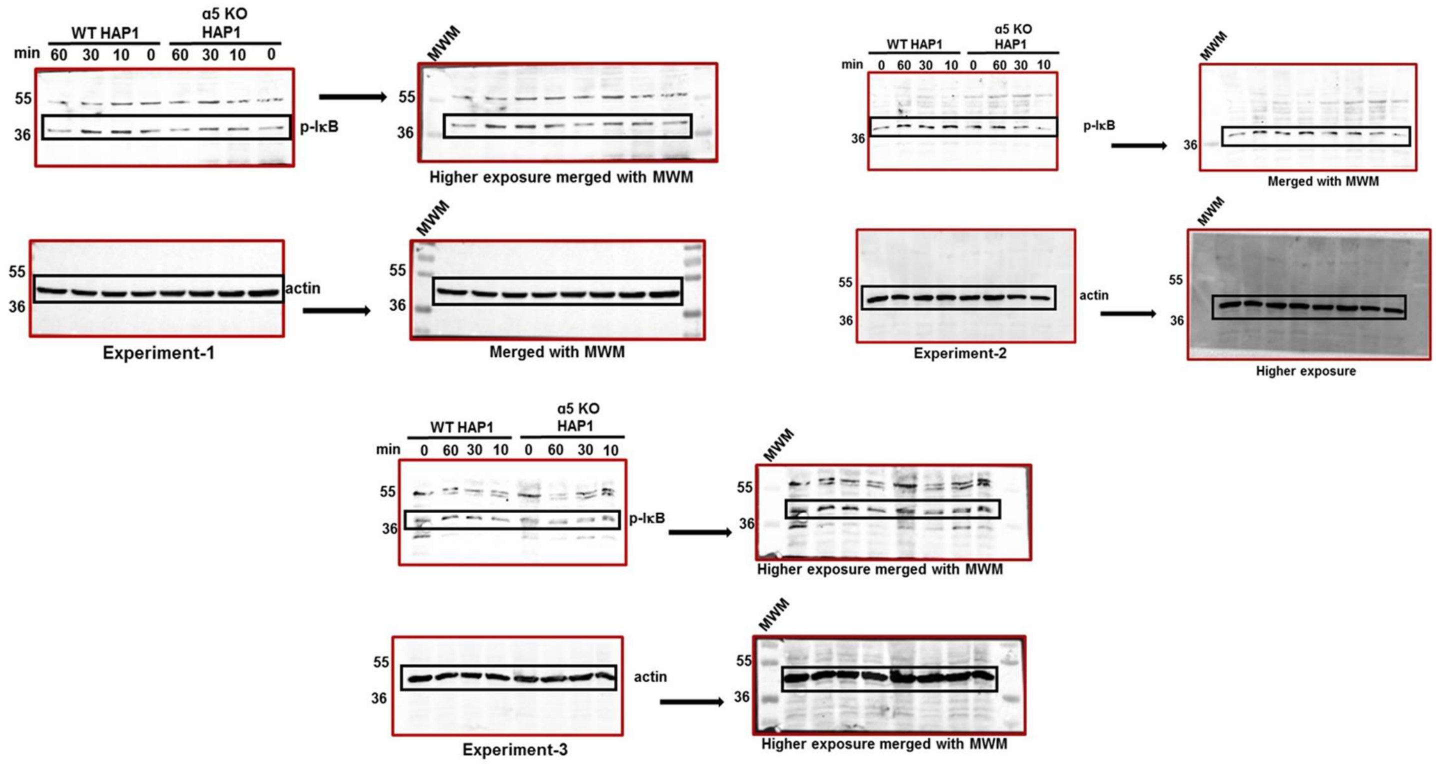

Supplementary Figure 10. **Uncropped Western blots corresponding to the figures in the manuscript.**

Supplementary Figure 10f. Western blots corresponding to Figure. 5d. The blot shown in experiment-1is displayed in Figure. 5d. Blots shown in experiment-2 and experiment-3 are independent repeats of the same experiment. Red and black outlines indicates uncropped and cropped images, respectively. For this experiment, after transfer, the membrane was cut into strips corresponding to the size of p-IκB and actin. The strips were then incubated with corresponding antibodies. Densitometric analyses shown in Fig. 5d was performed based on the quantification of the protein bands shown in experiment-1, experiment-2 and experiment-3. In some blots higher exposure is shown so that molecular weight marker (MWM) impression is visible. In addition, some blots were merged with MWM with or without higher exposure.

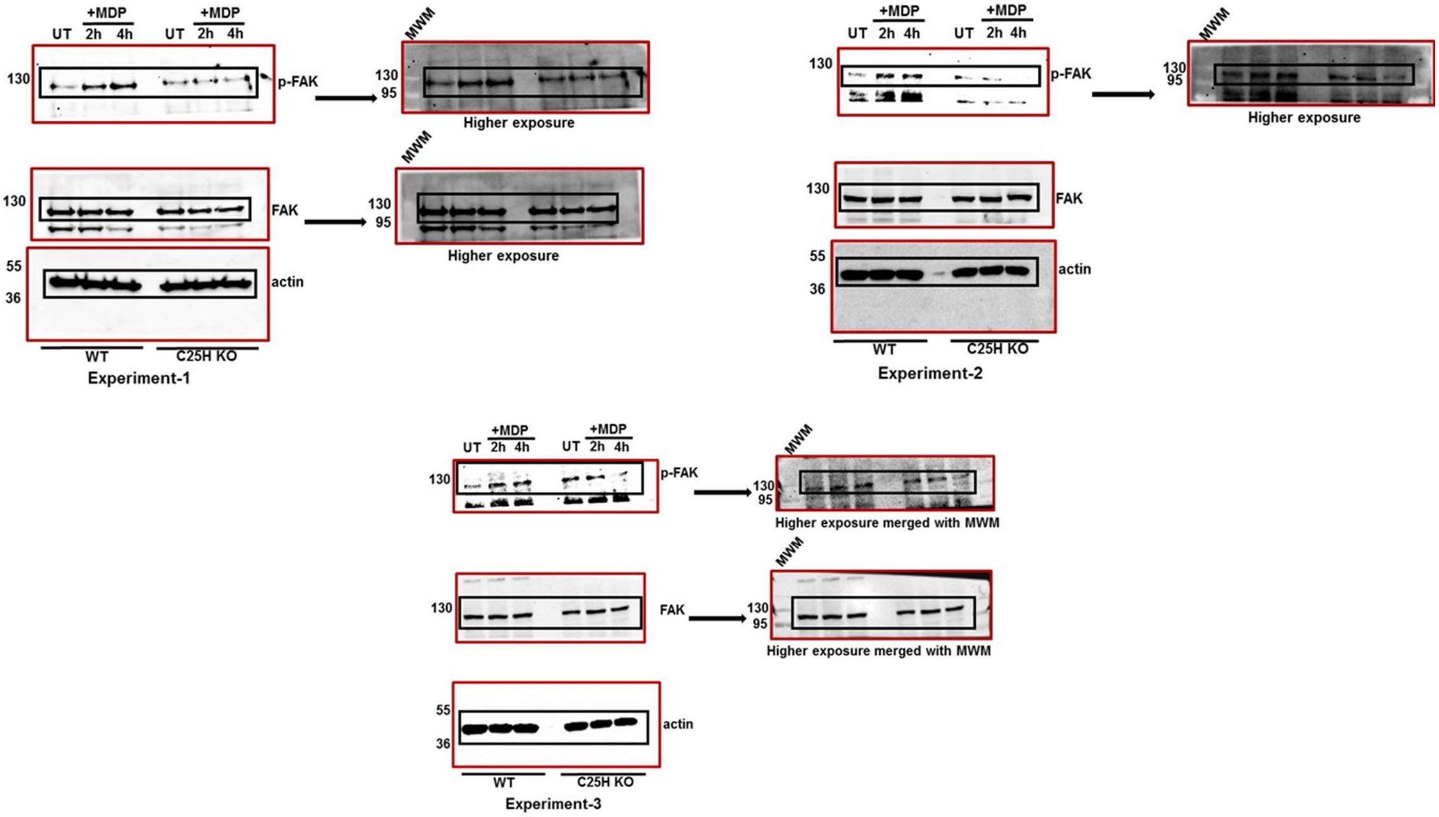

Supplementary Figure 10. **Uncropped Western blots corresponding to the figures in the manuscript.**

Supplementary Figure 10g. Western blots corresponding to Figure. 6m. The blot shown in experiment-1is displayed in Figure. 6m. Blots shown in experiment-2 and experiment-3 are independent repeats of the same experiment. Red and black outlines indicates uncropped and cropped images, respectively. For this experiment, after transfer, the membrane was cut into strips corresponding to the size of p-FAK, FAK and actin. The strips were then incubated with corresponding antibodies. Densitometric analyses shown in Fig. 6m was performed based on the quantification of the protein bands shown in experiment-1, experiment-2 and experiment-3. In some blots higher exposure is shown so that molecular weight marker (MWM) impression is visible. In addition, some blots were merged with MWM with higher exposure.

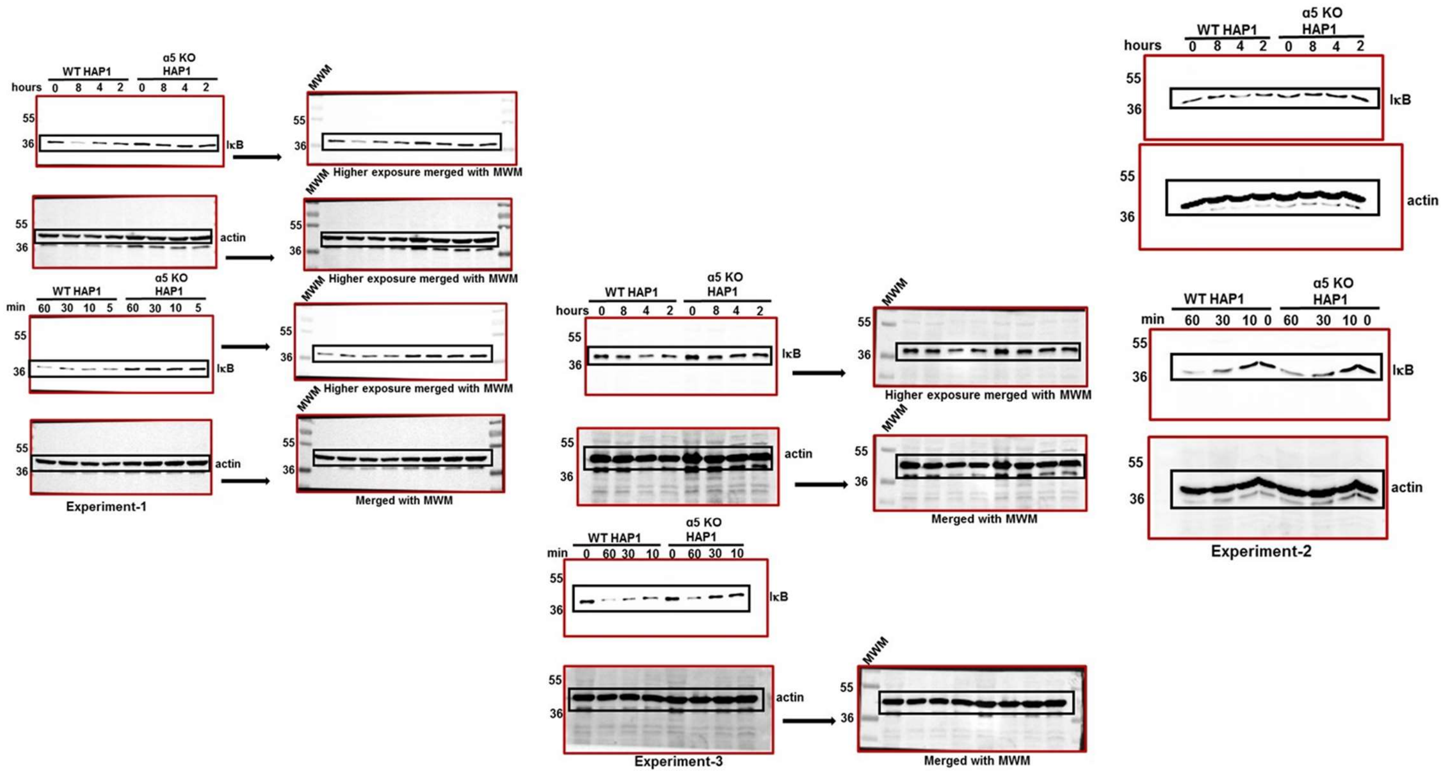

Supplementary Figure 10. **Uncropped Western blots corresponding to the figures in the manuscript.**

Supplementary Figure 10h. Western blots corresponding to Supplementary Figure. 5d. The blot shown in experiment-1is displayed in Supplementary Figure. 5d. Blots shown in experiment-2 and experiment-3 are independent repeats of the same experiment. Red and black outlines indicates uncropped and cropped images, respectively. For this experiment, after transfer, the membrane was cut into strips corresponding to the size of IκB and actin. The strips were then incubated with corresponding antibodies. Densitometric analyses shown in Supplementary Fig. 5d was performed based on the quantification of the protein bands shown in experiment-1, experiment-2 and experiment-3. Some blots were merged with molecular weight marker (MWM) with or without higher exposure.

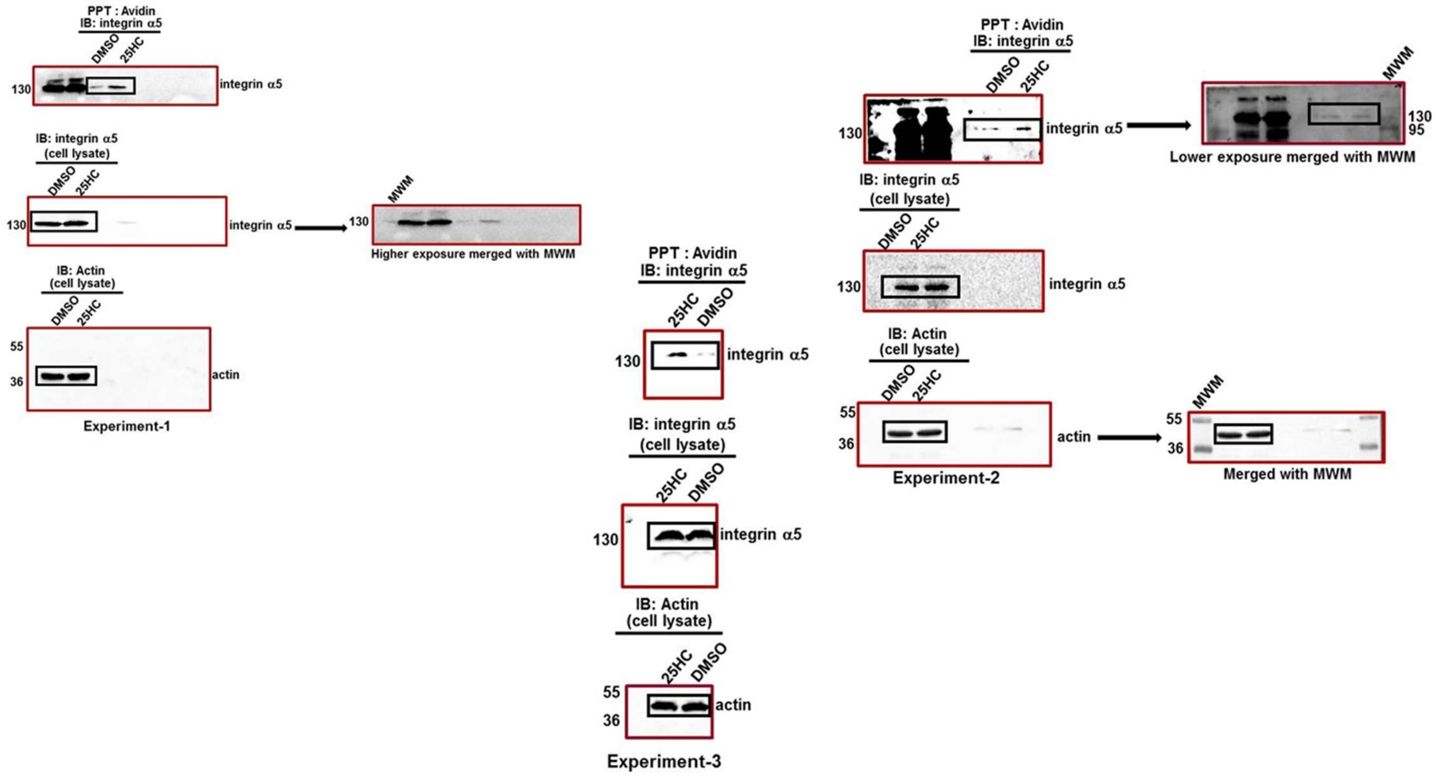

Supplementary Figure 10. Uncropped Western blots corresponding to the figures in the manuscript.

Supplementary Figure 10i. Western blots corresponding to Figure. 2a. The blot shown in experiment-1is displayed in Figure. 2a. Blots shown in experiment-2 and experiment-3 are independent repeats of the same experiment. Red and black outlines indicates uncropped and cropped images, respectively. For this experiment, after transfer, the membrane was cut into strips corresponding to the size of α5 integrin and actin. The strips were then incubated with corresponding antibodies. Some blots were merged with molecular weight marker (MWM) with or without higher or lower exposure.

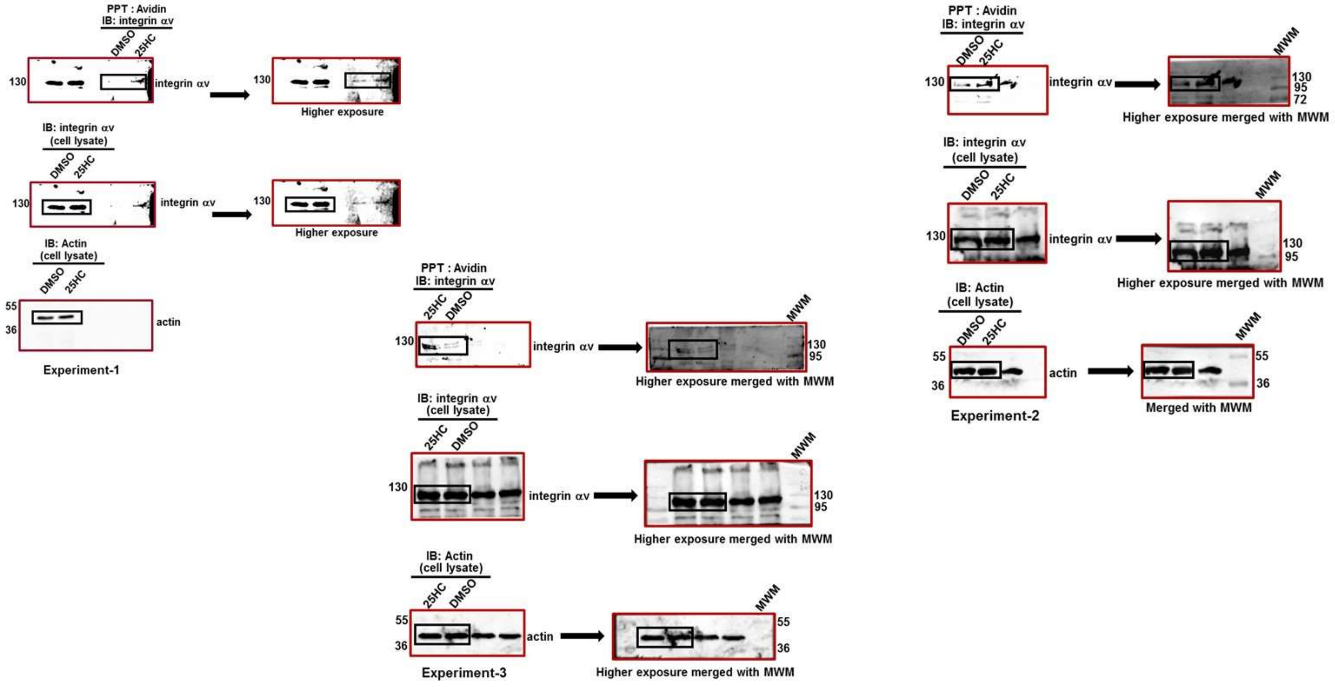

Supplementary Figure 10. **Uncropped Western blots corresponding to the figures in the manuscript.**

Supplementary Figure 10j. Western blots corresponding to Figure. 2b. The blot shown in experiment-1is displayed in Figure. 2b. Blots shown in experiment-2 and experiment-3 are independent repeats of the same experiment. Red and black outlines indicates uncropped and cropped images, respectively. For this experiment, after transfer, the membrane was cut into strips corresponding to the size of αv integrin and actin. The strips were then incubated with corresponding antibodies. Some blots were merged with molecular weight marker (MWM) with or without higher exposure

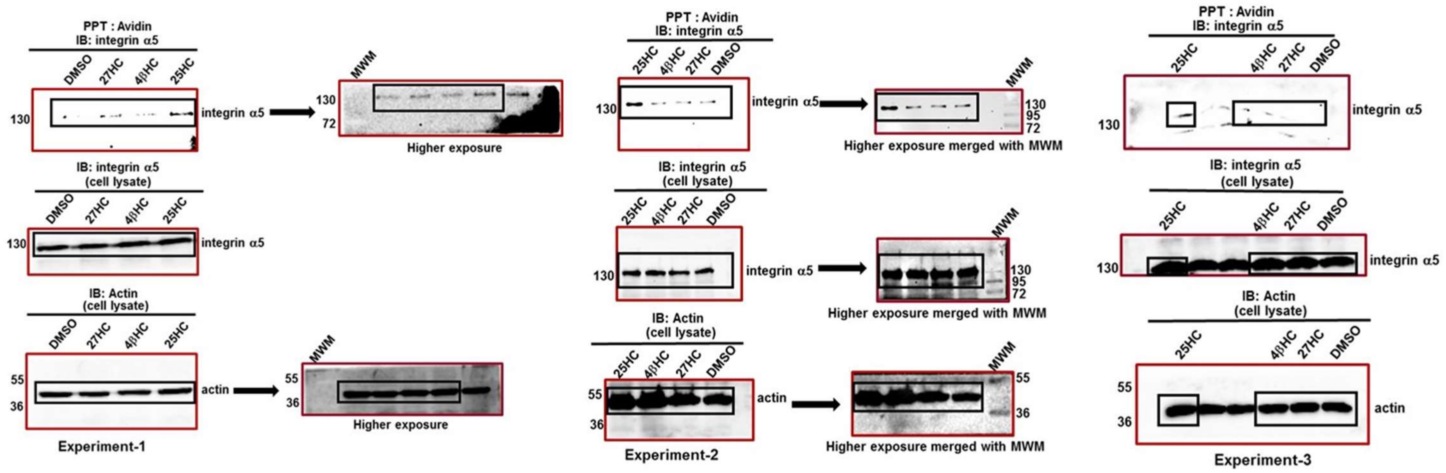

Supplementary Figure 10. **Uncropped Western blots corresponding to the figures in the manuscript.**

Supplementary Figure 10k. Western blots corresponding to Figure. 2g. The blot shown in experiment-1is displayed in Figure. 2g. Blots shown in experiment-2 and experiment-3 are independent repeats of the same experiment. Red and black outlines indicates uncropped and cropped images, respectively. For this experiment, after transfer, the membrane was cut into strips corresponding to the size of α5 integrin and actin. The strips were then incubated with corresponding antibodies. In some blots higher exposure is shown so that molecular weight marker (MWM) impression is visible. In addition, some blots were merged with MWM with higher exposure.

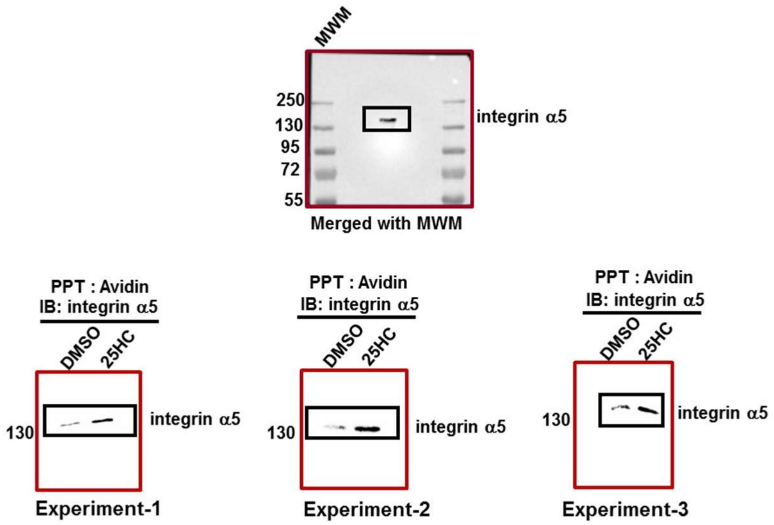

Supplementary Figure 10. **Uncropped Western blots corresponding to the figures in the manuscript.**

Supplementary Figure 10l. Western blots corresponding to Figure. 2c. TOP. Purified α5β1 integrin protein was subjected to SDS-PAGE. After transfer the membrane was cut horizontally into two portions (the upper and lower portions). The upper portion (i.e. above the 55 kDa molecular weight marker or MWM) was blotted with α5 integrin antibody. Only one band corresponding to α5 integrin was visible. Therefore, in subsequent Western blots related to *in vitro* experiments with purified α5β1 integrin (please see below), the membrane were horizontally cut into two portions following transfer and the top portion was blotted with α5 integrin antibody. BOTTOM. The blot shown in experiment-1is displayed in Figure. 2c (*in vitro* interaction of 25HC with purified α5β1 integrin protein). Blots shown in experiment-2 and experiment-3 are independent repeats of the same experiment. Red and black outlines indicates uncropped and cropped images, respectively. The blots were processed as described above.

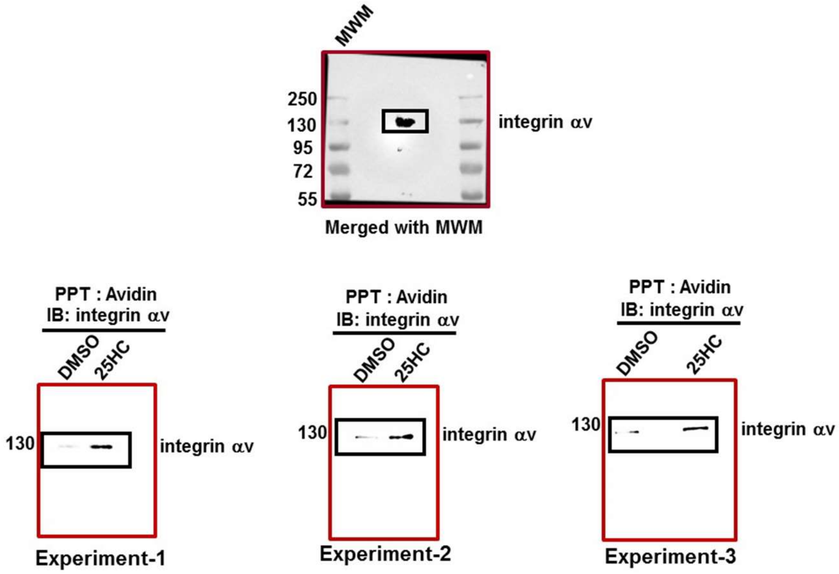

Supplementary Figure 10. **Uncropped Western blots corresponding to the figures in the manuscript.**

Supplementary Figure 10m. Western blots corresponding to Figure. 2d. TOP. Purified αvβ3 integrin protein was subjected to SDS-PAGE. After transfer the membrane was cut horizontally into two portions (the upper and lower portions). The upper portion (i.e. above the 55 kDa molecular weight marker or MWM) was blotted with αv integrin antibody. Only one band corresponding to αv integrin was visible. Therefore, in subsequent Western blots related to *in vitro* experiments with purified αvβ3 integrin (please see below), the membrane were horizontally cut into two portions following transfer and the top portion was blotted with αv integrin antibody. BOTTOM. The blot shown in experiment-1is displayed in Figure. 2d (*in vitro* interaction of 25HC with purified αvβ3 integrin protein). Blots shown in experiment-2 and experiment-3 are independent repeats of the same experiment. Red and black outlines indicates uncropped and cropped images, respectively. The blots were processed as described above.

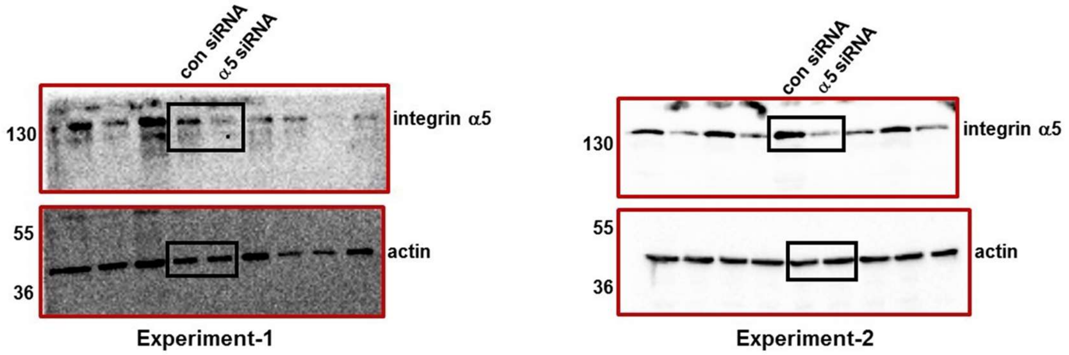

Supplementary Figure 10. **Uncropped Western blots corresponding to the figures in the manuscript.**

Supplementary Figure 10n. Western blots corresponding to Figure. 5b. The blot shown in experiment-1is displayed in Figure. 5b. Blot shown in experiment-2 is an independent repeat of the same experiment. Red and black outlines indicates uncropped and cropped images, respectively. For this experiment, after transfer, the membrane was cut into strips corresponding to the size of α5 integrin and actin. The strips were then incubated with corresponding antibodies. In some blots higher exposure is shown so that molecular weight marker (MWM) impression is visible. In addition, some blots were merged with MWM.

Supplementary Table 1. Lactate dehydrogenase (LDH) cellular cytotoxicity analysis (percent cytotoxicity)

Treatments	WT cells (MEFs)	FAK KO cells (MEFs)	Control cells (macrophages treated with vehicle)	Macrophages treated with FAK inhibitor
Positive control	100	100	100	100
25HC	0.66	0	2.85	2.44
MDP	0.28	0.14	1.92	2.91
RSV	0	0	3.93	4.48
IAV	0.59	0.25	1.02	0

Supplementary Table 1. LDH cellular cytotoxicity assay was performed with wild type (WT) and FAK knockout (KO) MEFs (mouse embryo fibroblasts) infected with virus (RSV or IAV) or treated with 25HC or MDP. A similar assay was also performed with primary immortalized mouse bone marrow derived macrophages (NR-9456 cells) treated with either vehicle (control cells) or FAK inhibitor during infection and 25HC or MDP treatment. As a positive control, cells were treated with a supplier provided agent that induces cellular toxicity. The values represent percent cytotoxicity.

Supplementary Table 2. Amino acid residues corresponding to 'RGD' binding site (site I) and 'non-RGD' binding site (site II) of βI and β-propeller domains of αvβ3 integrin

Binding Site	Binding Site Residues	
	**αv – β propeller domain**	**β3 – βI domain**
**Site I**	D150, Y178, Q180, T212, A213, Q214, A215, D218	S121, Y122, S123, R214, N215, R216, D217, A218, E220
**Site II**	E15, Y18, K42, N44, G49, I50, V51, E52, N77, S90, H91, W93, R122, A397, R398, S399, M400	H91, H113, V161, S162, A263, G264, I265, Q267, G276, S277, D278, N279, H280, S282, A283, T285, T286

Supplementary Table 3. List of PCR primers

Genes	Forward primer	Reverse primer
Human GAPDH	5′-GTCAGTGGTGGACCTGACCT	5′-AGGGGTCTACATGGCAACTG
Mouse GAPDH	5′GCCAAGGTCATCCATGACAACTTTGG	5′-GCCTGCTTCACCACCTTCTTGATGTC
Human TNF	5’-GGCAGTCAGATCATCTTCTCGAA	5’- GAAGGCCTAAGGTCCACTTGTGT
Human IL-6	5’-TACCCCCAGGAGAAGATTCC	5’- TTTTCTGCCAGTGCCTCTTT
Mouse C25H	5′-CCCATCTTCCCAGTCACCTT	5′-CAGGTGCTGGTAGAGTGTCA
Human α5 integrin	5’-CATTTCCGAGTCTGGGCCAA	5’-TGGAGGCTTGAGCTGAGCTT
Influenza A virus hemagglutinin	5’-CCCAAGGAAAGTTCATGG	5’-GAACACCCCATAGTACAAGG
Respiratory syncytial virus glycoprotein	5’- GCAGCAACAATCCAACCTGCTGG	5’- ATCGGAGGAGGTTGAGTGGAGGG

Supplementary Notes

Molecular interactions of α5β1 integrin and 25HC

Molecular docking and MD simulation study. We also investigated the potential molecular interactions and dynamics of 25-hydroxycholesterol (25HC) with α5β1 integrin by molecular docking and subsequent 200 ns long MD simulations of the bound complex, following the same methods described for αvβ3. The high resolution (2.5 Å) crystal structure of α5β1[1] integrin (PDB ID 4WK2) containing only the headpiece (β-propeller and βI subunits) was obtained from the Protein Data Bank. Our objective was to investigate whether the potential binding mode and molecular interactions of 25HC with α5β1integrin is similar to that of αvβ3 integrin-25HC interaction. Based on the size and complexity of the entire ectodomain of integrins, we did not try to model the missing knee and leg regions of the protein. Therefore, we restricted our analyses to binding interactions rather than seeking any conformational changes due to possible activation.

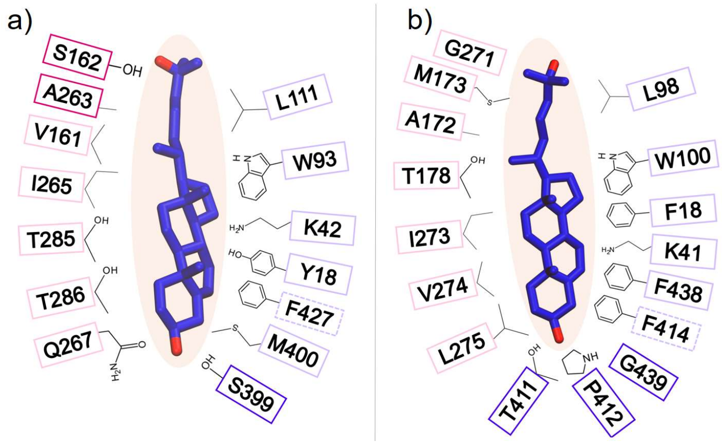

Fig. 1. Schematic representation of analogous binding modes of 25HC at site II of the integrins αvβ3 (a) and α5β1 (b) along with residues within 3.5Å distance from 25HC. The binding site residues from the β-propeller and βI domains are depicted in purple and pink boxes respectively. Residues involving H-bond interactions are depicted in dark shade boxes. The residues (F427 of αvβ3 and F414 of α5β1) interacting from the bottom distal region are depicted in dotted boxes.

Comparison of binding site (site II) residues and binding modes. Both αvβ3 and α5β1 integrins share a notable sequence identity and similarity among their binding site (site II) residues (Supplementary Note Fig. 1). As mentioned before, site II is distinct from the characteristic "RGD" motif binding site (site I) and is approximately 6 Å apart from the site I. Site II has been shown to bind to ligands and result in integrin activation. Recently published study[2] demonstrated that stromal cell-derived factor 1 (SDF1), a chemokine, which plays an important role in leukocyte arrest, binds to the site II and activate αvβ3 and α5β1integrins. Site II of αvβ3 and α5β1 integrins have several conserved residues that engage in the molecular recognition of 25HC. The conserved residues K42, L111, W93, and F427 of β-propeller domain of αvβ3 integrin correspond to K41, L98, W100, and F414 of α5β1 integrin, respectively. The aromatic amino acid Y18 of αvβ3 integrin corresponds to α5β1 integrin's F18 residue that lacks the -OH polar group. Site II interface of α5β1 integrin's βI domain is formed by residues A172, M173, T178, G271, I273, V274, and L275 whereas the corresponding interface in αvβ3 integrin is formed by residues V161, S162, A263, I265, Q267, T285, and T286.

Critical binding site interactions. Even though the binding modes of 25HC within these two pockets are very similar, the critical polar interactions observed in these two binding modes differ significantly. In αvβ3 integrin, both S162 and A263 from the βI domain participated in strong H-bond interactions with the 25-OH group of 25HC. Whereas in α5β1integrin, the M173 and G271 (Supplementary Note Fig. 1) engage in relatively very weak H-bond interactions through their backbone carbonyl and amide NH functional groups with the 25-OH of 25HC. The overall occupancy of these H-bonds were less than 20% of the entire 200 ns simulations. In contrast, three major H-bond interactions were observed at the 3-OH end of the 25HC molecule (distal end of the site II), which engaged with relatively stronger polar interactions with the residues T411, P412, and G439, characterized by their distance and angle (Supplementary Note Fig. 2a and 2b). The H-bond between the P412 –NH and 3-OH group is the strongest (with H-bond distance of < 3.5 Å and H-bond angle of ~150˚) for more than three-fourth of the simulation time. The electrostatic interactions between residues G439 and T411 have reasonable bond distance (~3.9 Å) and bond angle (~100 ˚), characteristics of moderately strong H-bond interactions.

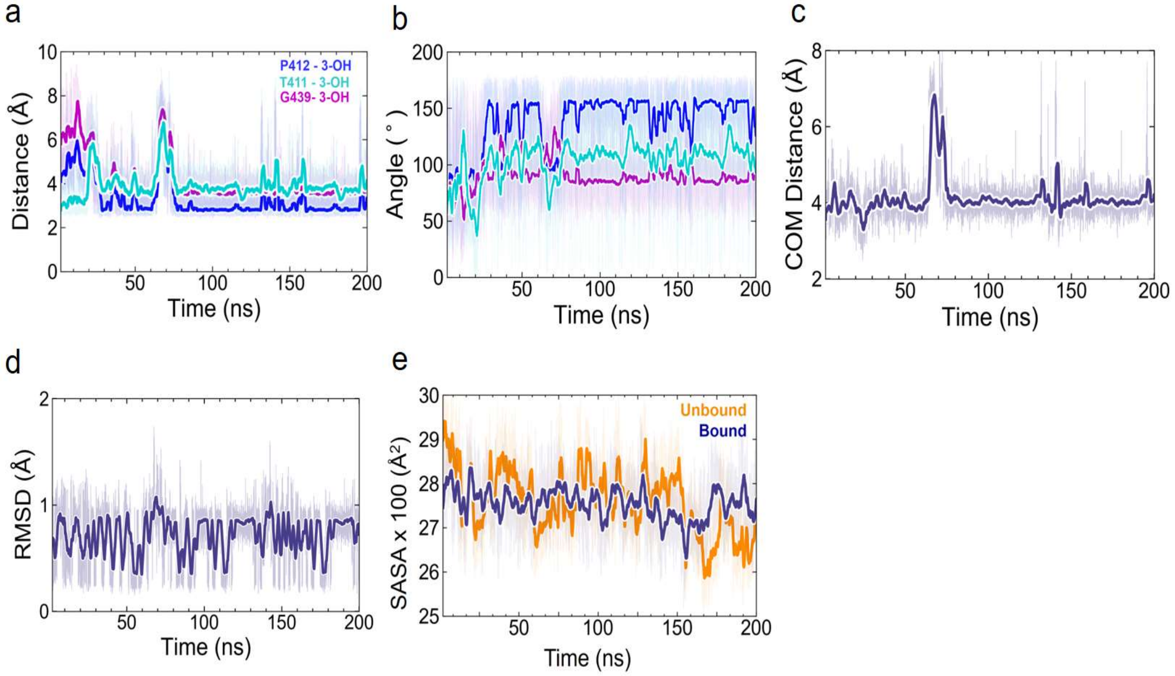

Fig. 2. Structural and dynamic observables in the molecular recognition of α5β1 integrin by 25HC: a) the H-bond distances and b) the H-bond angles between 3-OH of 25HC and the binding site residues Thr411(cyan) and Pro412 (blue), and G439 (magenta) respectively; c) Distance between center of mass of all binding site residues and that of 25HC atoms, indicating stable van der Waal (steric) interactions between these two molecules throughout the simulation; d) Root mean square deviation (RMSD) of 25HC within the binding site during stimulation time; e) Solvent accessible surface area (SASA) of the SDL loop (residues 168-198 of βI domain) in 25HC bound (blue) and unbound (orange) state.

In addition, it is important to note that the contribution of hydrophobic interactions seem to play a more critical role than that of polar interactions as evidenced by the number of hydrophobic residues (F18, L98, W100, F414, F438 of β-propeller domain and A172, I273, V274, and L275 of βI domain) surrounding the 25HC molecule, including the phenylalanine residues forming the base of the binding pocket. The strength of these hydrophobic interactions contributed by van der Waal (steric) forces can be qualitatively described by the distance (Supplementary Note Fig. 2c) between the center of mass

(COM) of all binding site residues and that of 25HC atoms, which is found to be ~4 Å for the majority of the simulation time. The root-mean square deviation calculated for the 25HC molecule showed minimal intramolecular fluctuations (RMSD between 0.4 to 0.8 Å) perhaps due to the characteristically rigid steroidal ring.

Specificity-determining loop. The binding of 25HC at site II of αvβ3 integrin resulted in significant conformational changes in the specificity-determining loop (SDL) as discussed in the manuscript's "Results" section. However, our root mean square fluctuation (RMSF), RMSD, and solvent-accessible surface area (SASA) calculations of the α5β1-25HC complex during the 200-ns simulation time did not reveal any significant conformational changes (Supplementary Note Fig. 2d) in the SDL. It is important to note that unlike αvβ3 integrin, in which the SDL and β-propeller blades engage in strong electrostatic interactions (please see Figures 3h and 3i of the main text), there is no such interaction seen in the crystal structure of α5β1 integrin. The significant conformational change noted only in the 25HC-bound αvβ3 integrin resulted due to the breakage of these electrostatic interactions. Upon further structural analyses, it was observed that the SDL loop of α5β1 integrin has some α-helical content that is absent in the SDL loop of αvβ3. The presence of additional secondary structural constraints might be the reason for the lower flexibility of SDL during interaction of 25HC with α5β1 integrin. In addition, the absence of strong H-bond interaction from the SDL residues (Ser162 of αvβ3 integrin made a strong H-bond at the 25-OH end of 25HC) may explain the absence of any such triggering effect within the simulated time. It should be noted that unlike the αvβ3-25HC model, the simulation of the α5β1-25HC complex model suffers from the absence of knee and leg regions and further investigation with the complete structure upon availability is warranted.

The movie depicting the trajectory from the 200 ns MD simulations of the α5β1integrin-25HC complex can be found in Supplementary Movie 4.

Supplementary References:

1. Xia, W. & Springer, T.A. Metal ion and ligand binding of integrin alpha5beta1. *Proc. Natl. Acad. Sci.* **111**, 17863-17868 (2014).
2. Fujita, M., Davari, P., Takada, Y.K. & Takada, Y. Stromal cell-derived factor-1 (CXCL12) activates integrins by direct binding to an allosteric ligand-binding site (site 2) of integrins without CXCR4. *Biochem. J.* **475**, 723-732 (2018).
