# Supplementary material for: Integrin activation by the lipid molecule 25-hydroxycholesterol induces a proinflammatory response
Source: Nat Commun. 2019 Apr 1;10:1482. doi: 10.1038/s41467-019-09453-x (PMC6443809; doi:10.1038/s41467-019-09453-x)
Supplement: Supplementary file 3 — Description of Additional Supplementary Files [file 41467_2019_9453_MOESM3_ESM.pdf]

Description of Additional Supplementary Files

Supplementary Movie 1.
Trajectory from the first 50 ns of total of 100 ns MD simulations of αvβ3 integrin-25HC complex in which the ligand was initially docked to the classical 'RGD' binding site (site I). The 25-OH oxygen was predicted to interact with the Mn^{2+} ion in the initial docked pose, but the interaction became extremely weak within the first 2 ns of the simulation and resulted in 25HC drifting away from the metal ion. During these 50 ns, the 25HC molecule was seen surveying the neighboring territories of site I but it did not recognize any stable and strong interactions

Supplementary Movie 2.
Trajectory from the 200 ns MD simulations of αvβ3 integrin-25HC complex in which the ligand was docked to site II. The H-bond interactions between the two OH groups of 25HC and Ser399 of β-propeller, Ser162 of βI domains of αvβ3 integrin remained intact for almost the entire simulation time duration. The H-bond between Ala263 of the βI domain and 25-OH of the ligand was observed to break around 110 ns as the ligand readjusts its orientation within the binding site. The breaking of electrostatic interactions between the β-propeller loops and specificity determining loop (SDL) results in significant conformational change that starts around ~30 ns.

Supplementary Movie 3.
Porcupine plot analyses of 25HC-bound αvβ3 integrin. The direction and extent of motions observed at the genu (knee) site and between the head and leg regions indicate increased interactions between these regions during the simulation.

Supplementary Movie 4.
Trajectory from the 200 ns MD simulations of the α5β1integrin-25HC complex (secondary structure representation of β-propeller and βI domains are in blue and magenta, respectively) in which the ligand (in licorice representation and blue color) was bound to site II. The binding site residues (M173, G271, T411, P412, and G439) engaged in H-bond interactions with 25HC are highlighted in licorice representation and cyan color. Unlike αvβ3 integrin (Supplementary Movie 2), no interactions between the SDL and β-propeller loops of α5β1integrin was observed during the simulation.
